# Inertia-driven amphibious robot with asymmetric microundulatory fin arrays

**DOI:** 10.1126/sciadv.aea2222

**Published:** 2026-02-18

**Authors:** Lingqi Tang, Yongzun Yang, Bing Li, Bingfu Zhang, Qiguang He, Hongliang Ren, Yao Li

**Affiliations:** ^1^ School of Robotics and Advanced Manufacture, Harbin Institute of Technology, Shenzhen, Shenzhen, China.; ^2^ Mechanical and Automation Engineering Department, The Chinese University of Hong Kong, Hong Kong, China.; ^3^ Electronic Engineering Department, The Chinese University of Hong Kong, Hong Kong, China.; ^4^ Guangdong Provincial Key Laboratory of Intelligent Morphing Mechanisms and Adaptive Robots, Shenzhen, China.; ^5^ Key University Laboratory of Mechanism & Machine Theory and Intelligent Unmanned Systems of Guangdong, Shenzhen, China.; ^6^ State Key Laboratory of Robotics and Systems, Harbin, China.

## Abstract

Centimeter-scale amphibious robots are promising for versatile tasks. Existing solutions use active and multiple mechanisms for environmental interaction; however, such designs face sealing challenges at small scales and are often complex and unreliable. Here, we present an inertia-driven actuation strategy combining a variable-output voice coil motor (VCM) with a fully sealed rigid shell. By modulating the VCM output, the robot achieves jumping, full-stroke vibration for terrestrial locomotion and small-stroke vibration for aquatic propulsion. Terrestrial tests demonstrate rapid motion on granular media, continuous jumping, and load carrying. The robot also uses passive tilted fins that convert reciprocating motion into steerable aquatic thrust, realizing an inertia-driven multidirectional propulsion mechanism. Thrust generation and frequency-dependent propulsion were analyzed through aquatic experiments, high-speed particle image velocimetry, and simulations. Last, a 24-gram legless prototype (Leglessbot) demonstrated effective locomotion across diverse terrain, offering a compact solution for underactuated amphibious mobility.

## Teaser

A compact robot uses an internal inertial actuator to crawl, jump, and perform steerable swimming.

## INTRODUCTION

Microrobots are gaining traction for their potential to perform inspection, environmental monitoring, and rescue tasks in confined or hazardous environments. Being small and agile, they can access areas that are difficult or unsafe for larger robots or humans to enter. Researchers have developed various locomotion strategies to enable microrobots to adapt to complex terrain, including legged (*1*), wheeled (*2*), tracked (*3*), rolling (*4*), and undulatory (*5*) mechanisms for terrestrial movement. In aquatic environments, propulsion is typically achieved using propellers, active fins (*6*), mechanical jets (*7*), or chemical jets (*8*). Despite this progress, most microrobots are designed to operate in a single type of environment. Achieving effective amphibious locomotion in both domains remains a major challenge, especially at a centimeter scale.

Researchers have explored various technologies to achieve effective amphibious locomotion with simple, minimal actuation, including amphibious wheels (*4*, *9*, *10*), pneumatic (*11*) and electrohydraulic (*12*, *13*) actuators, morphological legs (*14*, *15*), snake-like bodies (*16*), and rigid-flexible hybrid vibration modules (*17*). However, reliable waterproof sealing is a critical challenge in centimeter-scale robots. Conventional dynamic sealing structures (e.g., O-rings) become increasingly impractical at small scales because tolerance-induced gaps readily cause capillary ingress, and the required sealing friction forces may exceed the capabilities of miniature actuators. Recent works using either dual-chamber jet actuation (*7*) or fully soft, oil-immersed electronics (*18*) have circumvented these issues by avoiding mechanical transmission together to minimize sealing demands. Inspired by this trend, a fully enclosed body is a promising development direction for amphibious microrobots, as it offers considerable advantages in terms of reliability and durability across diverse conditions.

Inertia (e.g., linear, angular, and centrifugal inertia) is produced by moving parts inside the body. Inertia-driven robots have no active motion parts outside the body and can be fully sealed into a rigid shell. Recently, researchers have studied several methods for driving locomotion using inertia. The asymmetric linear momentum produced by a piezo actuator with different forward and backward speeds can effectively drive linear motion, such as pipe climbing (*19*). The symmetric linear momentum of a robot with an asymmetric footpad can also drive fast locomotion (*20*–*22*). Cubli (*23*) is driven by angular momentum from three flywheels and can balance on its tip and roll across various terrains. Using a similar approach, NASA’s Hedgehog (*24*) shows effectiveness in low-gravity space. An eccentric rotating mass (ERM) motor (*25*) can also produce centrifugal inertia, and the resulting asymmetric friction with flat ground can produce in-plane locomotion, such as in Kilobot (*26*), GASR (*27*), and Leafbot (*28*).

However, current inertia-driven robots can hardly jump or swim, limiting their working space. The reason lies in three aspects: First, the output of the inertia source encounters a trade-off between output momentum and frequency; second, one inertia source can only perform one type of motion, so adding another locomotion (e.g., jumping and swimming) will greatly increase the system size and weight; and last, inertia-driven aquatic locomotion is challenging because of the zero overall output inertia. Systematic study of inertia-driven techniques remains scarce.

In this study, a variable-output voice coil motor (VCM) was designed for multimode actuation, enabling the development of a 24-g inertia-driven legless robot (namely Leglessbot). The robot demonstrated high-impact resistance and rapid locomotion on granular media. Inspired by vascular valves that passively respond to flow, we designed tilted fins that interact asymmetrically with fluid during body vibration, enabling propulsion without active control. Steerable swimming was achieved by only one actuator, facilitating inertia-driven multidirectional propulsion (IDMP), a mechanism for planar swimming. Aquatic experiments and high-speed particle image velocimetry (PIV) measurements confirmed IDMP. Validated computational fluid dynamics (CFD) simulations of fluid-structure interaction further elucidated the thrust-generation mechanism of the passive fins and the frequency-dependent thrust behavior. Last, comprehensive amphibious tests confirmed the robot’s crawling, jumping, impact resistance, escaping from ballast, and steerable swimming capabilities, highlighting the substantial potential of inertia-driven techniques.

## RESULTS

### Locomotion design

**Fig. 1.**
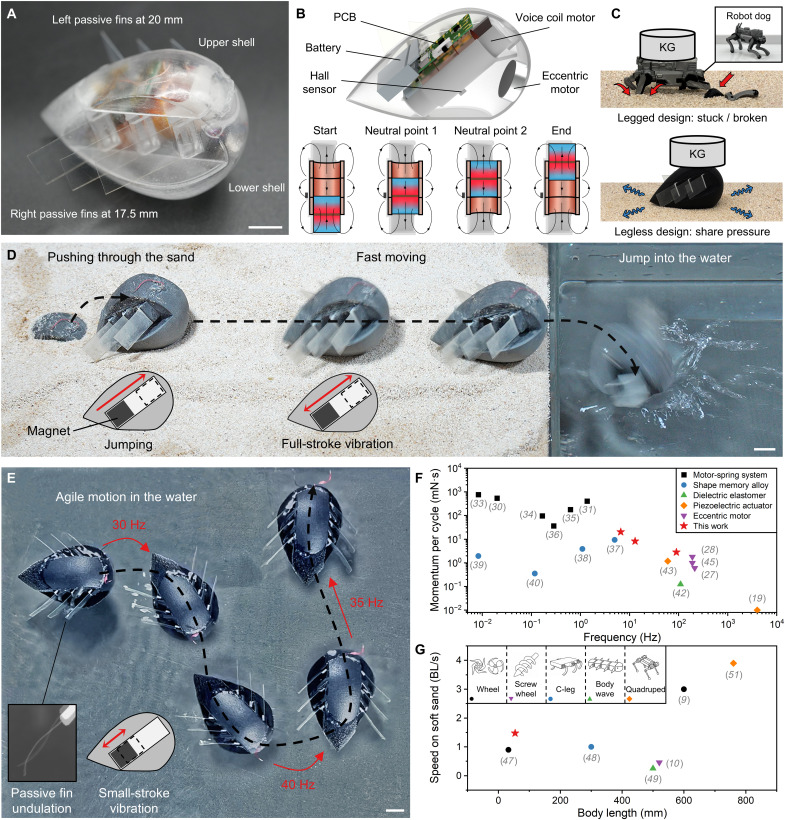
Proposed inertia-driven Leglessbot. (**A**) The upper and lower shells seal the inner components, and six soft, asymmetrical passive fins are arranged on the left and right sides. (**B**) Its simple yet effective inner components are a VCM with a Hall sensor, an ERM motor, a printed circuit board (PCB), and a battery. (**C**) Schematic diagram comparing legged and legless designs. (**D**) The robot leaps from the sand by jumping, moves quickly via full-stroke vibration, and jumps into the water. (**E**) Steerable swimming in small-stroke vibration mode is achieved by actuating the VCM at different frequencies, which causes varied responses from the left and right fins, enabling the robot to steer and swim forward. (**F**) Current actuation methods address the output momentum in terms of stroke and frequency. (**G**) The graph shows the walking speeds of existing self-contained robots on dry sand. Scale bars, 10 mm.

Leglessbot (Fig. 1A) was designed to traverse various terrains through simple actuation. As shown in Fig. 1B, the robot consists of a shell, passive fins of two lengths, a linear VCM, an ERM motor, a printed circuit board (PCB), and a battery. All components are glued together without conventional motion transmission (e.g., use of gears or linkages). The VCM is rigidly fixed to the hollow shell, which houses all components and includes internal ribs for structural reinforcement. This transmission-free design simplifies sealing and improves robustness by eliminating exposed moving parts. The VCM forms a 45° angle with the ground, maximizing the jumping distance. Because of its simple actuation, the robot only weighs 24 g and measures 57.5 mm by 36 mm by 36 mm. Figure 1C and movie S7 demonstrate the advantages of the legless design, where the enclosed shell facilitates pressure dispersion, and the absence of exposed or load-bearing appendages avoids getting stuck and vulnerability to damage.

Unlike conventional VCMs, the proposed VCM has a longer stroke and various vibration modes. First, the overall magnet consists of two magnets bonded with their N poles face to face to minimize disturbance from external magnetic fields (e.g., geomagnetic field). Three coils are placed close to each other and electrically connected; the two outer coils are connected in series with the same polarity, while the middle coil is wound in the opposite direction (see details in figs. S1 and S2). To actuate a full stroke (Fig. 1B), the magnet goes through the coil from the bottom to the top, with its location being monitored by the Hall sensor. When the magnet reaches a neutral point, that is, the position where the Lorentz force changes sign, the coil swaps its current direction—meaning that the driver reverses the coil current to maintain continuous acceleration of the magnet. This design can provide a large output momentum (20.2 mN·s) despite its compact size (Φ14 mm by 34 mm). When the coil swaps before it reaches a neutral point, the VCM vibrates quickly (>20 Hz) in a small stroke (<3 mm). Thus, three actuation modes can be achieved (Fig. 1, D and E, and movie S1): In jumping mode, coil swaps occur exactly at the neutral points, so the magnet traverses the full stroke and impacts the shell to launch the robot; in full-stroke vibration, successive swaps drive continuous large-amplitude oscillations for terrestrial locomotion; and in small-stroke vibration, earlier swaps confine the magnet motion to short oscillations but at a high frequency, which is advantageous for aquatic propulsion.

The robot can launch itself from sand in jumping mode, move fast [∼1.4 body lengths (BL)/s] in full-stroke vibration mode on dry sand, and swim in small-stroke vibration mode (Fig. 1, D and E). The soft, passive fins facilitate aquatic propelling and controllable steering motion, enabling planar swimming actuated by a single actuator. At 35 Hz, fins of both lengths have the same thrust, and the robot travels straight. At 30 and 40 Hz, the long-left fins (20.0 mm length) and short-right fins (17.5 mm length) dominate the motion, allowing the robot to turn right or left, respectively.

A benchmarking of actuation methods highlights the trade-off between output momentum and frequency in miniature robots (Fig. 1F): Motor-spring systems (*29*–*36*) deliver a high momentum (36 to 762 mN·s) but at a low frequency (<2 Hz); shape memory alloy systems (*37*–*40*) suit miniaturization but are power-inefficient; and dielectric (*41*, *42*), piezoelectric (*19*, *43*, *44*), or eccentric motors (*27*, *45*, *28*) reach higher frequencies (>100 Hz) but produce insufficient momentum (<2 mN·s) for explosive jumping. The proposed variable-output VCM bridges this gap by generating 20.2 mN·s at 6.6 Hz for jumping, 8.2 mN·s at 13 Hz for ground launch, and 2.8 mN·s at 90 Hz for swimming. On challenging granular media, where yielding substrates impede motion and dust causes motor failure, large robots (>500 mm) rely on wheels (*9*, *10*, *46*, *47*), C-legs (*48*), body undulation (*49*, *50*), or quadrupeds (*51*), while small-scale solutions (<100 mm) require complex transmissions and achieve <1 BL/s. In contrast, our legless design has no exposed moving parts, enabling simple and robust locomotion at 1.4 BL/s on dry sand—faster than existing self-contained robots at a comparable scale (Fig. 1G).

### VCM and terrestrial performance characterization

**Fig. 2.**
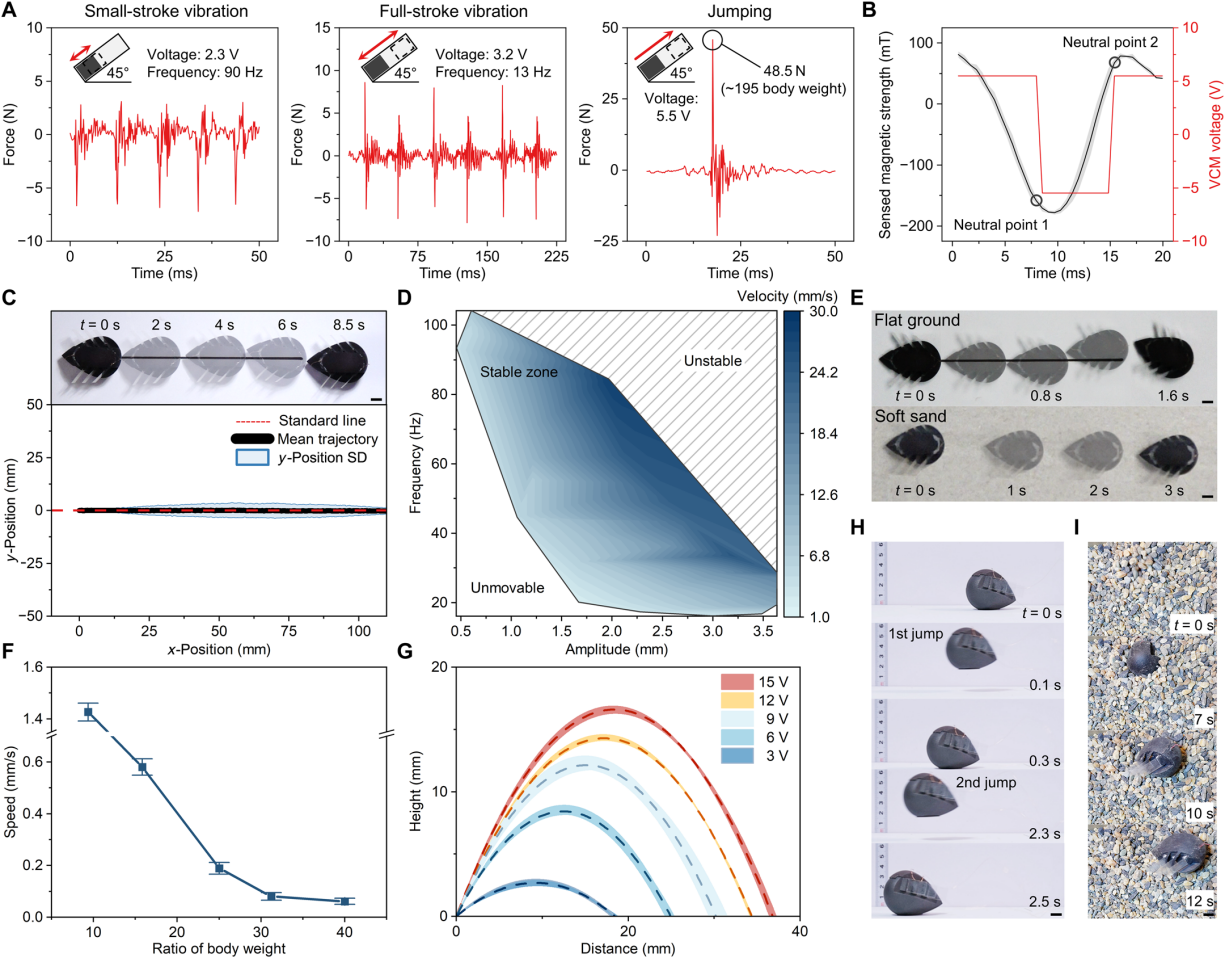
VCM and terrestrial tests. (**A**) The graphs show the VCM output forces in small-stroke vibration, full-stroke vibration, and jumping modes. (**B**) The graph depicts the variation in magnet strength during jumping and the VCM current direction swaps at neutral points 1 and 2. Error bars: standard deviation for *n* = 5 samples. (**C**) The robot performs highly precise (maximum standard deviation: 3.577 mm) forward motion on flat ground in small-stroke vibration mode. Error bars: standard deviation for *n* = 8 samples. (**D**) The forward motion speed in small-stroke vibration mode varies with the VCM frequency and amplitude. (**E**) The time-lapse images show the robot in small-stroke vibration and full-stroke vibration modes. (**F**) The robot has excellent weight capacity in jumping mode, moving forward while carrying a 960-g weight (~40 times its body weight). Error bars: standard deviation for *n* = 3 samples. (**G**) The adjustable jumping height varies with the VCM input voltage. The maximum jumping height is 17.16 mm (at 15 V). The average trajectories and profiles from three trials are shown for each voltage. (**H**) In jumping mode, the robot can continuously jump via direction actuation and (**I**) push through granular media. Scale bars, 10 mm.

Figure 2A characterizes the real-time output force in the three VCM modes: (i) In open-loop controlled small-stroke vibration mode, the coils are powered at 2.3 V and 90 Hz to induce small, quick vibrations, resulting in asymmetric force profiles with a dominant negative peak per cycle. (ii) In open-loop controlled full-stroke vibration mode (at an overall frequency of 13 Hz), the coils are driven at 3.2 V to prevent overheating, allowing the magnet to oscillate between the top and bottom of the shell, producing alternating peak forces with each collision. (iii) In feedback jumping mode (with a maximum frequency of 6.6 Hz), the coils are fully powered (5.5 V by the battery and 15 V by an external power source) to pull the magnet to the top rapidly, generating a strong impact force of up to 45.8 N. The force curves in three modes are all asymmetrical because the VCM forms a 45° angle with the ground. Figure 2B shows the magnetic field measured by the Hall sensor as the magnet keeps going upward in jumping mode. The coil current switches at the two neutral points (−150 and 70 mT) for continuous actuation.

In small-stroke vibration mode, the robot’s trajectory on a hard surface (Ethylene-Vinyl Acetate plate, hardness factor: 60°) has good accuracy, with a maximum trajectory standard deviation of 3.577 mm (Fig. 2C). In this mode, the speed of the robot’s straightforward motion can be varied by changing the magnet vibration amplitude and frequency. The highest straightforward speed of 28.1 mm/s (∼0.5 BL) is achieved at a VCM frequency of 80 Hz and an amplitude of 0.858 mm. Figure 2D shows the vibration frequency-amplitude-velocity relationship in small-stroke vibration mode. At a small magnet vibration amplitude and frequency, the robot cannot move forward because of the insufficient momentum generated by the VCM. On the contrary, at an excessively high amplitude and frequency, the robot’s motion becomes violent, and the trajectory is uncontrollable. In the region in the figure where the robot can move stably, the robot’s motion speed is positively correlated with the product of the vibration frequency and amplitude of the VCM. This is because the magnet gains a higher momentum and undergoes more momentum exchanges, thereby driving the robot faster.

In full-stroke vibration mode, the robot performs excellently on granular media. Because of impact inertia, the robot is slightly lifted above the ground (∼5 mm) and then quickly lands; its legless design prevents it from plunging into the soft ground. As shown in Fig. 2E and movie S2, the robot can reach a forward speed of ∼78 mm/s (∼1.4 BL/s) on dry sand and 41.6 mm/s (∼0.7 BL/s) on flat ground.

The jumping mode differs from the conventional energy-storage-and-release process; the violent collision of the magnet can directly drive the whole robot to jump. The untethered VCM module provides a large load capacity (960 g, or ∼40 times the robot body weight) at a speed of 0.07 mm/s (Fig. 2F). The robot even moves and escapes under 5-kg weights (∼208 times its body weight; movie S7). Figure 2G plots the jump trajectory against the actuation voltage. The jump height can be changed by adjusting the input VCM voltage. The maximum jump height reaches 17.16 mm (∼0.5 body height) at a driving voltage of 15 V. With its “tumbler” design, the robot remains upright and jumps continuously (Fig. 2H and movie S4). The robot also excellently pushes itself through granular media, leaping out from a pile of round beads (with diameters of ∼5 mm) within 2 s and from a heap of small rocks (with diameters of ∼5 mm) within 10 s (Fig. 2I).

### Swimming performance and hydrodynamic mechanisms

#### Robot aquatic motion characteristics

**Fig. 3.**
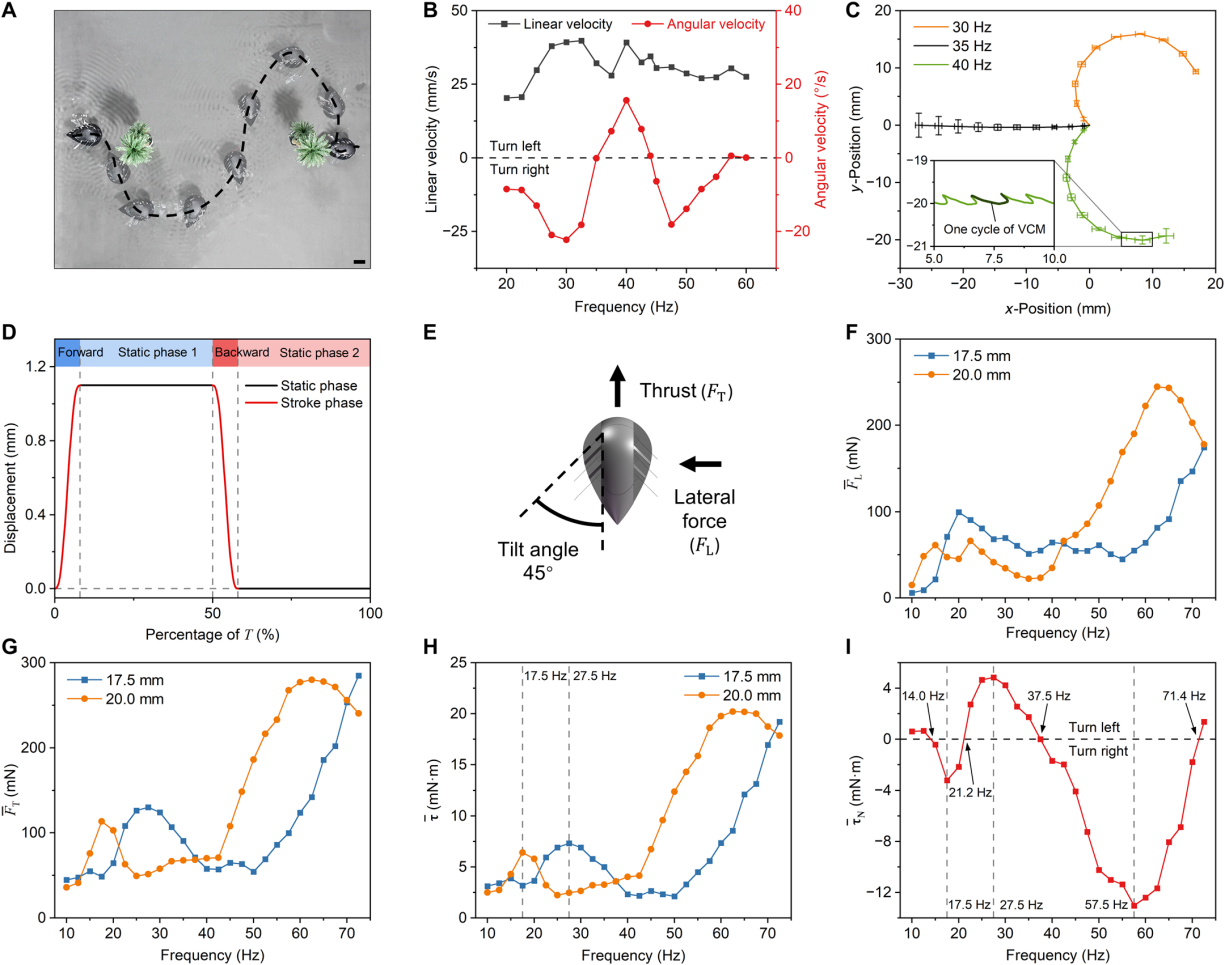
Aquatic tests and CFD results. (**A**) The snapshots show an “S” curve obstacle avoidance motion driven by the VCM at 30, 35, and 40 Hz. (**B**) Measured aquatic linear and angular velocity curves versus frequency. The angular velocity curve crossed zero three times, representing three straightforward frequencies. Error bars represent the standard deviation for *n* = 3 samples and are smaller than the symbols. (**C**) Repetitive trajectories of the robot aquatic motion at 30, 35, and 40 Hz, with terminal distribution circle radii of 0.47, 2.88, and 1.45 mm, respectively. Error bars: standard deviation for *n* = 3 samples. The trajectory consists of the difference between the forward and backward displacements in each cycle. (**D**) Simulated square-wave displacement of the VCM vibration. Each cycle consists of forward stroke, static phase 1, backward stroke, and static phase 2. (**E**) Orientation definition of the vector symbols. Each fin was installed at an angle of 45°. (**F** to **H**) Time-averaged CFD results for fins of both lengths versus frequency: (F) lateral force $\bar{F_{L}}$, (G) thrust $\bar{F_{T}}$, and (H) torque $\bar{\tau }$ . (**I**) The simulated time-averaged net torque-frequency curve intersected with the zero value four times, showing a notable similarity to the angular velocity-frequency profile in (B). Scale bar, 20 mm.

The prototype demonstrated IDMP in water using its asymmetric fins. The VCM vibration frequency was tuned for rapid directional control and flexible swimming. Figures 1E and 3A show the robot’s agile steering movements with a minimum radius of 5.6 mm. Straight swimming was observed at 35, 44, and 60 Hz at speeds of ∼32, 35, and 28 mm/s, respectively (Fig. 3B). IDMP was achieved as follows: The robot turned rightward at 20 to 35 and 44 to 60 Hz (peak: 22°/s at 30 Hz) and leftward at 35 to 44 Hz (peak: 16°/s at 40 Hz).

The robot’s aquatic motion was agile and had high trajectory consistency. The terminal distribution circle radii for the 30-, 35-, and 40-Hz triple-repeat trajectories were 0.47, 2.88, and 1.45 mm, respectively (Fig. 3C). Moreover, the overall trajectory consisted of periodic microdisplacements in the forward and backward directions. In each vibration cycle, the forward displacement of the robot was slightly larger (~1.2 mm) than the backward displacement, resulting in overall forward movement.

These kinematic observations characterized IDMP, but the underlying hydrodynamic mechanisms required further investigation. Force or pressure sensors could not be used to measure the hydrodynamic load of the inertia-driven prototype directly, as they would have changed the system’s dynamic response. Thus, the hydrodynamic principle of IDMP was studied by conducting PIV measurements to characterize the flow field, along with a detailed analysis based on CFD.

The CFD simulation focused on the fluid-fin interaction dynamics, so the inertia output was simplified to a square wave with a fixed amplitude of 1.1 mm (Fig. 3D), which was obtained from a high-speed video camera. Here, thrust ($F_{T}$) is positive in the robot’s forward direction, the lateral force ($F_{L}$) is positive from the outside toward the robot, and the torque (τ) from the left fins is positive for rightward steering, and vice versa (Fig. 3E). The VCM vibrating frequency is denoted by f, and T is the cycle period of the system. Transient values are indicated by the original letters ($F_{T}$), and time-averaged values have bars ($\bar{F_{T}}$).

The thrust, lateral force, and torque strongly depended on frequency and length-induced misalignment (Fig. 3, F to H). Fins of both lengths (17.5 and 20.0 mm) exhibited synchronized dual-peak trends in $\bar{F_{T}}$ and $\bar{\tau }$ against f, whereas $\bar{F_{L}}$ had a weaker bimodal pattern. The $\bar{F_{T}}$ and $\bar{\tau }$ profiles were strongly linked because the robot’s rotation center was in the middle of the three evenly spaced fins on each side, which meant that the sideway forces canceled each other’s torque. From 10 to 40 Hz, the $\bar{F_{T}}$ of the 17.5- and 20.0-mm fins reached initial peaks of 130 and 113 mN at 17.5 and 27.5 Hz, corresponding to $\bar{\tau }$ values of 7.3 and 6.4 mN·m, respectively. A further increase in f reduced $\bar{F_{T}}$ and $\bar{\tau }$. Beyond 40 Hz, the 20.0-mm fin showed a second peak (280 mN at 62.5 Hz, $\bar{\tau }$ = 20.2 mN·m), and the 17.5-mm fin showed a sustained rise in $\bar{F_{T}}$ (285 mN at 72.5 Hz, $\bar{\tau }$ = 19.2 mN·m). This bimodal pattern resembles the amplitude-frequency responses of cantilever beams; the first peaks (27.5 and 17.5 Hz) aligned with first-mode resonance, and the subsequent peak (62.5 Hz) corresponded to second-mode resonance. In this work, only IDMP at lower frequencies was studied because the fins exhibited complex bending and torsion movements in higher-order regions (>50 Hz), making analysis difficult.

f considerably influenced the time-averaged net torque ($\bar{\tau_{N}}$), calculated by subtracting the $\bar{\tau }$ produced by the fins on both sides, which determined the robot’s steering. Define $\bar{\tau_{N}}$ that makes the robot turn left as positive. As shown in Fig. 3I, $\bar{\tau_{N}}$ reached zero four times, indicating that the robot had four straightforward swimming frequencies (14, 21, 37.5, and 71 Hz), three left-turn frequency intervals, and two right-turn frequency intervals between 10 and 72.5 Hz. This simulated curve agreed well with testing results (Fig. 3B), confirming the macroscopic correctness of the numerical simulations. However, substituting displacement for the inertia-driving process in the simulation, the material coefficients (e.g., Young’s modulus and Poisson’s ratio), or prototype manufacturing errors may result in a certain offset between simulations and experiments.

However, the time-averaged results did not capture details inside every cycle. To investigate the IDMP mechanism, the following analysis focuses on the transient results and explores the principles of thrust generation, frequency effects, and the emergence of straightforward frequencies.

#### Principle of aquatic thrust generation

**Fig. 4.**
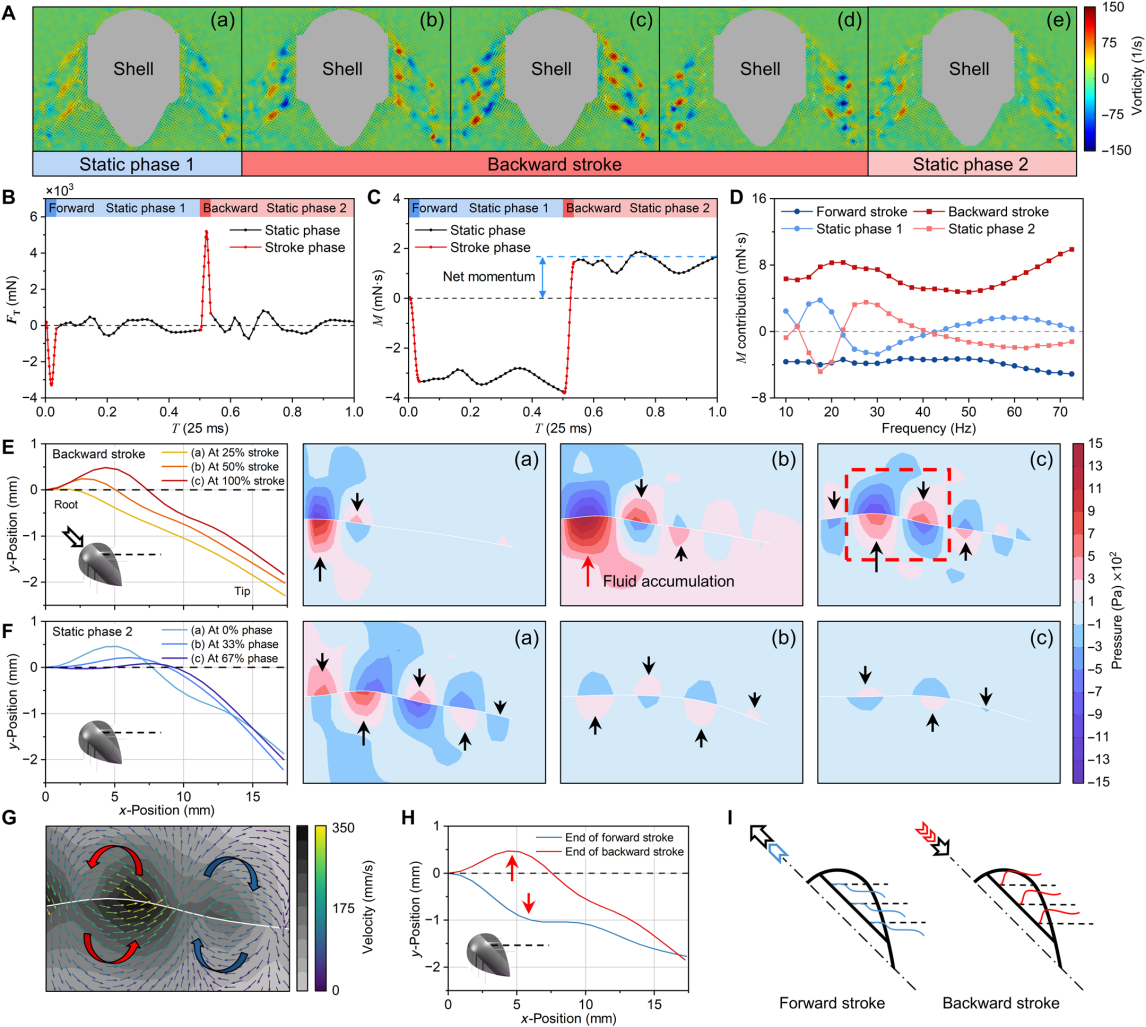
Aquatic thrust generation and transient characteristics. (**A**) PIV-measured continuous transient vorticity fields at 30 Hz. The backward stroke generated alternating-sign vortex structures propagating outward along the fins (>150 s^−1^). Vorticity was weak (<60 s^−1^) in the static phases, indicating that the fluid interacted with the fins mainly in the stroke phases. The asymmetric vorticity field on both sides suggested that the fins on the two sides interacted differently with the fluid at the same frequency, resulting in steering motion. (**B** and **C**) Transient thrust and momentum curves in one vibration cycle for a typical case (17.5-mm fin at 40 Hz). The thrust and momentum changed considerably (−3.38 and +5.14 mN·s) in the stroke phases. (**D**) Momentum contributions of the four phases in one vibration cycle at all frequencies. The net momentum mainly came from the momentum difference of the two stroke phases, as static-phase contributions basically canceled out. (**E** and **F**) The graphs and images show the fin shapes and corresponding pressure fields during the backward stroke and static phase 2. The solid arrows show the local thrust direction. (**G**) Local magnification of the velocity fields in [E(c)]. The vortices captured via PIV consisted of annular flows on both sides of the fin without any connection, showing that the principle of IDMP is different from that of fish. (**H**) The fin shapes at the end of the forward and backward strokes showed that the tilted placement of the fins resulted in different sizes of the fluid accumulation zones, as indicated by the red arrows. (**I**) The schematic diagrams show the shapes of the fins as the robot made stroke motions. During the forward impact of the robot, the fins shrank to reduce forward resistance; during the backward impact, the fins opened to increase backward resistance.

Three typical frequencies were analyzed in the PIV experiment: 30 Hz (maximum rightward steering), 35 Hz (straightforward propulsion), and 40 Hz (maximum leftward steering). Figure 4A presents the transient vorticity fields at 30 Hz, including a complete stroke cycle (see the complete process at all three frequencies in movie S10). In the backward stroke phase, a distinctive vortex propagation pattern characterized by sequential generation and directional alternation emerged at the fins. High-intensity (~140 s^−1^) vortices formed near the fin root [Fig. 4A(b)] and propagated distally along the fin surface, exhibiting counterrotating configurations between adjacent vortex structures [Fig. 4A(c)]. Before and after the backward stroke, the vortices had weak intensities (~50 s^−1^) [Fig. 4A, (a) and (e)]. These hydrodynamic signatures revealed that the fins interacted more strongly with the water during the stroke phases than in the static phases. The vorticity fields at the fins on both sides were simultaneously asymmetric, suggesting that the fins of both lengths interacted differently with the water at the same frequency, giving the robot a steering behavior (which is further analyzed in the next section).

**Fig. 5.**
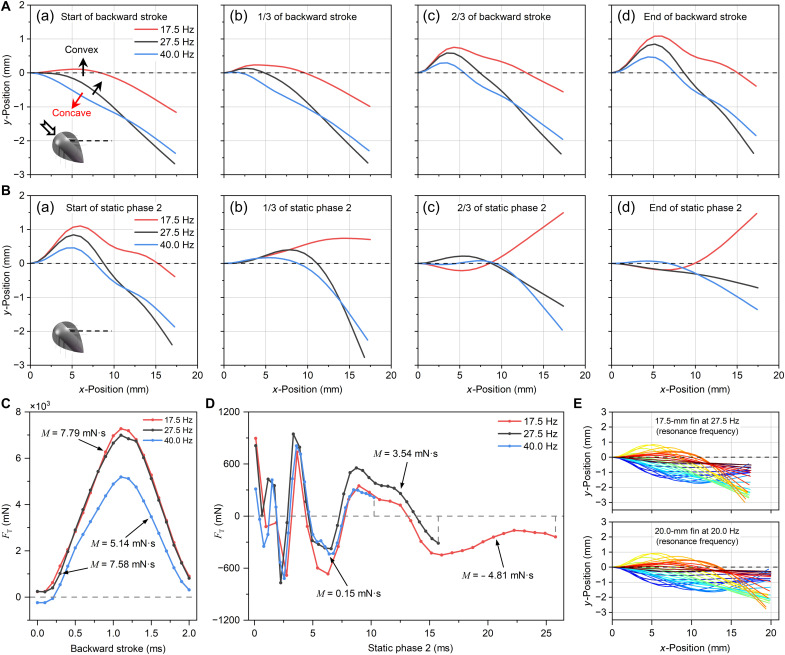
Effect of frequency on the thrust and emergence of straight-line frequencies. (**A** and **B**) The graphs depict the shapes of the 17.5-mm fin in the backward stroke and static phase 2 at the three typical frequencies: 17.5, 27.5, and 40 Hz. (**C**) The transient force curves of the 17.5-mm fin in the backward stroke show that the 40-Hz fin had a smaller final momentum because of the negative initial value and smaller slopes. (**D**) The transient force curves of the 17.5-mm fin in static phase 2 indicate that the damping process was interrupted at different times because of the frequency change, resulting in a different final momentum and the initial value for the next stroke. (**E**) The graphs show similar vibration profiles at resonance for both fin lengths. Because of their length difference, the vibration pattern of the 20-mm fin lagged behind that of the 17.5-mm fin overall by 7.5 to 10 Hz. This lag reflects the translation of the $F_{T}$-𝑓 curves in Fig. 4G, with the intersection producing a straightforward frequency.

The net moment accumulation mainly arose from the differential contributions between the forward and backward strokes. Figures 4B and 5C show the variations in $F_{T}$ and the corresponding momentum (M), respectively, from a 17.5-mm fin vibrating at 40 Hz throughout one cycle, as calculated via CFD. The cycle was divided into four distinct phases, namely, forward stroke, static phase 1, backward stroke, and static phase 2, corresponding to the square-wave displacement input, aligned with Fig. 3D. The thrust exhibited pronounced variations between motion phases. During the stroke phases, the thrust rapidly varied, with peak magnitudes approaching 5 N, inducing substantial momentum changes (−3.38 and +5.14 mN·s). By contrast, the static phases showed constrained thrust oscillations within ±0.8 N about the neutral axis, resulting in negligible momentum (−0.47 and +0.15 mN·s). Thus, the overall net momentum in one cycle was 1.44 mN·s, produced from the difference between the forward stroke and the backward stroke (Fig. 4C). This characteristic was consistent across all frequencies (from 10 to 72.5 Hz), as evidenced in Fig. 4D, where the momentum (M) contribution source was divided into the four phases. The contributions from the backward stroke were larger than those from the forward stroke (>1.46 mN·s), and the oscillatory components during the static phases exhibited nearly mutual cancellation.

The difference in thrust between the four phases stemmed from variations in the fluid-solid behavior. The pressure field and fin shape (Fig. 4, E and F) and the correlated velocity field (movie S9) were analyzed to determine the reasons for the generation of the thrust. In the fin profiles in Fig. 4 (E to I), the black dashed line is the undeformed fin. In the pressure field figures (Fig. 4, E and F), the solid arrows represent the local thrust direction, which aligns with the local projection direction of the fin.

During the backward stroke, the fluid-structure interaction caused strain concentration and structural fluctuation propagation, thereby inducing transient thrust generation and decay. Initially, the fin root moved by 1.1 mm because of inertial actuation, and the fin pushed the water. The resistance of the water also forced the fin to deform and created symmetrical positive and negative pressures on each fin side [Fig. 4E(a)]. The short stroke duration (2 ms) induced an exceptionally high velocity (∼550 mm/s) at the fin root, leading to a high hydrodynamic load (∼1580 Pa) in this critical region [Fig. 4E(b)]. Meanwhile, the transient nature of the stroke caused the distal region to maintain motion inertia; concentrated deformation occurred at the root, resulting in localized fluid accumulation [red arrow in Fig. 4E(b)], which further increased the pressure. This fluid-structure interaction mechanism simultaneously amplified both the strain concentration and hydrodynamic pressure at the fin root. The location of the severe deformation in Fig. 4E(b) caused other parts to deform to share the stress, so the wave peak moved outward from the root (nature of the structural mechanism), forming alternating peaks and valleys [Fig. 4E(c)]. The wave peaks and valleys exhibited opposite thrusts, and their forces offset each other, decreasing the overall thrust to only 0.7 N.

The transient pressure and velocity fields during the stroke phase corresponded directly to the PIV-captured pseudovortices, confirming that the propulsion principle differed from that of fish-tail propulsion. Figure 4G presents the locally amplified velocity fields in Fig. 4E(c). The PIV-captured transient pseudovortices were actually “assembled” from the annular flows. These pseudovortices resembled units but were divided by the fin (white line); each unit “consists” of two centrally symmetrical annular flows on either side of the fin. The essence of circulation was the fluid squeezing-backfilling effect produced during the transmission of fluctuations: The peaks (convex surfaces) squeezed the fluid outward (from the root to the tip), and the fluid backfilled the valleys (concave surfaces), building transient-like pseudovortices as they moved. Thus, the pseudovortex positions corresponded one to one to the local thrust points of the fins. Although the pseudovortices exhibited a superficial morphological analogy to the von Kármán vortex streets generated by fish-tail motion, they fundamentally varied in formation mechanisms and hydrodynamic coupling, confirming that their thrust-generation principle fundamentally differed from the propulsion mechanism of fish. Crucially, the congruence between the PIV-observed pseudovortices and the CFD-resolved annular flows validated the transient-level accuracy of the CFD simulations.

Following the stroke phase, the static phase was governed by elastic restitution and hydrodynamic dissipation, which had a much lower contribution to the overall momentum (minimum of 3% at 35 Hz and maximum of 40% at 10 Hz). During the initial relaxation, the residual fluid momentum maintained a measurable pressure (∼700 Pa) across the fin surfaces [Fig. 4F(a)]. However, with the cessation of the external excitation and the progressive dissipation of the stored elastic energy in the fin, the fluid pressure decayed markedly to ∼200 Pa [Fig. 4F(b)]. Consequently, the pressure field—exhibiting quadratic dependence on the flow velocity—decreased rapidly, from ~150 Pa [Fig. 4F(c)] to less than 50 Pa. The transient force curves of the fins repeated around the zero value in the static phase, as shown by the black line in Fig. 4B. This was because, during wave propagation, alternating generations of wave peaks and valleys produced opposing thrust directions. When a wave peak was transmitted to the terminal end and released its energy, the valleys dominated the thrust direction, shifting it to negative values. Subsequently, as a wave valley was transmitted to the terminal end and dissipated, the increased proportion of peaks reversed the thrust to positive values, establishing a cyclical alternation. A comparative analysis of Fig. 4F [(a) to (c)] demonstrates direction alterations in local thrust at identical fin positions, exhibiting continuous variation patterns aligned with elastic rebound phenomena. Because of the alternating dominance of positive/negative thrust phases and the progressively diminishing magnitudes from damping effects, the net momentum increment (0.15 mN·s) became substantially lower than that observed during the stroke phase (5.14 mN·s). This oscillatory behavior sharply contradicted the unidirectional accumulation characteristic of the stroke phase.

The stroke phases produced a net momentum because of the tilted installation of fins, whereas the static phases had a negligible overall contribution (fig. S5). Figure 4H illustrates the fin profiles at the end of the forward and backward strokes. During a forward stroke, hydrodynamic resistance restricted fin advancement, generating backward-directed thrust ($F_{T}\leq 0$). The fins retracted to mitigate fluid drag while reducing the fluid accumulation in the root deformation zone. During backward impact, hydrodynamic forces opposed the fin’s backward movement, producing a forward-directed thrust ($F_{T}\geq 0$). Fin opening amplified the resistance and enhanced the fluid accumulation in the root deformation zone. This augmented backward resistance manifested as proportionally increased forward thrust generation. According to a comparative analysis of fin morphologies, despite the equivalent stroke displacement and time, backward strokes resulted in a greater fluid accumulation area, with more severe structural deformation (red arrows in Fig. 4H). This geometric disparity enhanced the local pressure differentials by at least 28%, consequently generating a net momentum. As illustrated in Fig. 4I, this difference lastly produced a net thrust and displacement in each cycle, as also confirmed by the microscopic trajectory in Fig. 4C.

#### Principle of IDMP

In this study, robotic propulsion relied on the momentum differentials between forward and backward strokes. Both experiments and simulations consistently demonstrated that the fin’s time-averaged thrust markedly depended on frequency. The thrust combinations of the left and right fins were changed by adjusting the frequency, lastly facilitating IDMP. This section contains a detailed analysis of the principle of the observed frequency-dependent thrust generation.

First, the fundamental mechanisms underlying the $\bar{F_{T}}$-f relationship in fixed-length fins are elucidated. Three characteristic frequencies within the first resonant frequency interval (10 to 50 Hz) of the 17.5-mm fin were selected for analysis: the peak thrust frequency (27.5 Hz) and the two flanking minima (17.5 and 40 Hz). Given the trend symmetry between the forward (static phase 1) and backward (static phase 2) strokes, Fig. 5 (A and B) illustrates the fin deformation evolution during the backward stroke and static phase 2 at these frequencies (see the complete process in movie S9). The corresponding transient thrust profiles are presented in Fig. 5 (C and D) (see pressure fields in movie S9).

In each cycle, beginning with a backward stroke, the frequency affects the initial form of the fin, which mainly affects the thrust amplitude. As shown in Fig. 5A [(a) to (d)], during the backward stroke, the fin roots exhibited considerable deformation at the three characteristic frequencies, and the deformation patterns were geometrically similar. This explained the consistent thrust variation trend in Fig. 5C. However, the 40-Hz case exhibited a negative initial thrust (positive for 17.5/27.5 Hz) because of its reverse prebending configuration [red arrow in Fig. 5A(a)] relative to the forward bending at the other frequencies (black arrows). On the basis of the established principle, the local thrust direction aligned with the fin convex orientation (detailed pressure fields in movie S9), and the 40-Hz case required fluid pressure to counteract the negative initial thrust before generating an upward-curving positive thrust. This led to persistently smaller deformations [Fig. 5A, (c) and (d)] and weaker fluid accumulation at 40 Hz, as indicated by the reduced growth slopes of the thrust curve. Consequently, the 40-Hz thrust profile shifted downward overall, yielding a ∼32% lower momentum contribution than at the other frequencies. Therefore, in the stroke phases, the initial fin geometry served as a determinant, as it preset the initial thrust vector polarity (positive or negative) and modulated the thrust growth slopes through deformation-induced fluid accumulation. This geometric preconditioning was inherently governed by the fin shape at the end of the preceding static phase.

The frequency adjustment changed the duration of the static phases that were dominated by the damped motion. Thus, the fin entered the next stroke in different damping stages at various frequencies. The thrust curves at the three frequencies were similar but were cut off at different times (Fig. 5D). The static time of the 17.5-Hz fin was sufficient (Δt=26.6 ms), allowing for a relatively complete fluctuation—the fin rebounding from convex bending [Fig. 5B(a)] to the neutral state [Fig. 5B(b)] and then bending back in the opposite direction after energy release [Fig. 5B(c/d)]. However, because of the concave rebound, the generated net momentum was negative (−4.81 mN·s). The 27.5-Hz fin had a moderate static time (Δt=16.2 ms), completing only the forward energy release. The fin reset to the neutral state [Fig. 5B(d)], achieving efficient positive momentum accumulation (+3.54 mN·s). The 40-Hz fin had an insufficient static time (Δt=10.5 ms), forward deformation was not fully released, and the fin retained residual strain energy [Fig. 5B(d)], with a negligible net momentum (+0.15 mN·s). From an efficiency perspective, the 17.5-Hz fin released more energy but mainly produced negative thrust (∼72% of static phase 2), the 40-Hz fin did not release enough energy because of its insufficient duration, and the 27.5-Hz fin released enough energy with high efficiency because it produced positive thrust most of the time (∼65% of static phase 2). Thus, the residence duration (frequency) governed the cutoff position of time, resulting in different ultimate signs and values of thrust and momentum. The initial form of the fin for the following stroke consequently differed.

In summary, the frequency varied the net momentum by changing the cutoff time of the static phase. This cutoff time affected the amount of momentum generated during this phase and determined the prerequisites for the next stroke phase. These prerequisites were the value of the initial thrust and the initial shape of the fin, which determined the amount of momentum generated in the stroke phase. The 27.5-Hz fin had the highest energy utilization in the static phase and provided good preparation for the next stroke. Thus, 27.5 Hz can be assumed as the resonant frequency. Before 27.5 Hz, the static phase was less energy-efficient; after 27.5 Hz, the preparation conditions in the stroke phase were inferior. According to the trend symmetry (Fig. 5D), the 40-Hz thrust cut off at positive values, whereas the 17.5- and 27.5-Hz ones cut off at negative values. These were the opposite of the initial values at the beginning of the backward stroke. For the next (forward) stroke, the fins continued following a similar symmetrical cycle, performing steady-state vibration.

Given the principle of frequency-dependent thrust (or momentum) in fixed-length fins, a combination of asymmetric fins can be used to achieve straightforward motion and steering or IDMP. According to vibration mechanics, beam parameters affect the resonant frequency. This also applies in wet mode, as shown by Ansys Workbench simulation results (fig. S6), although the resonant frequency of a beam decreases in the fluid. Here, the experiments showed that thrust reached a local maximum near the resonant frequency. Thus, designing fins of different lengths can obtain varying resonant frequencies. Furthermore, the region between adjacent peak frequencies of the left and right fins must have cross-points that make fins of different lengths produce the same thrust; on each side of the intersection frequency, the robot should have controlled opposite steering directions. As shown in Fig. 3G, the $\bar{F_{T}}$-f curves of two fins were geometrically similar but had a lateral translation. The intersection points that emerged were the straightforward ones (14.0, 21.2, 37.5, and 71.4 Hz). The vibration patterns of the 17.5- and 20-mm fins were highly similar at their respective first-order resonant frequencies (Fig. 5E), and the vibration patterns of the 20-mm fin generally lagged behind those of the 17.5-mm fin overall by 7.5 to 10 Hz (figs. S7 and S8). Therefore, fins of both lengths vibrated the same at the misaligned position, confirming the abovementioned translational theory.

### System demonstration

**Fig. 6.**
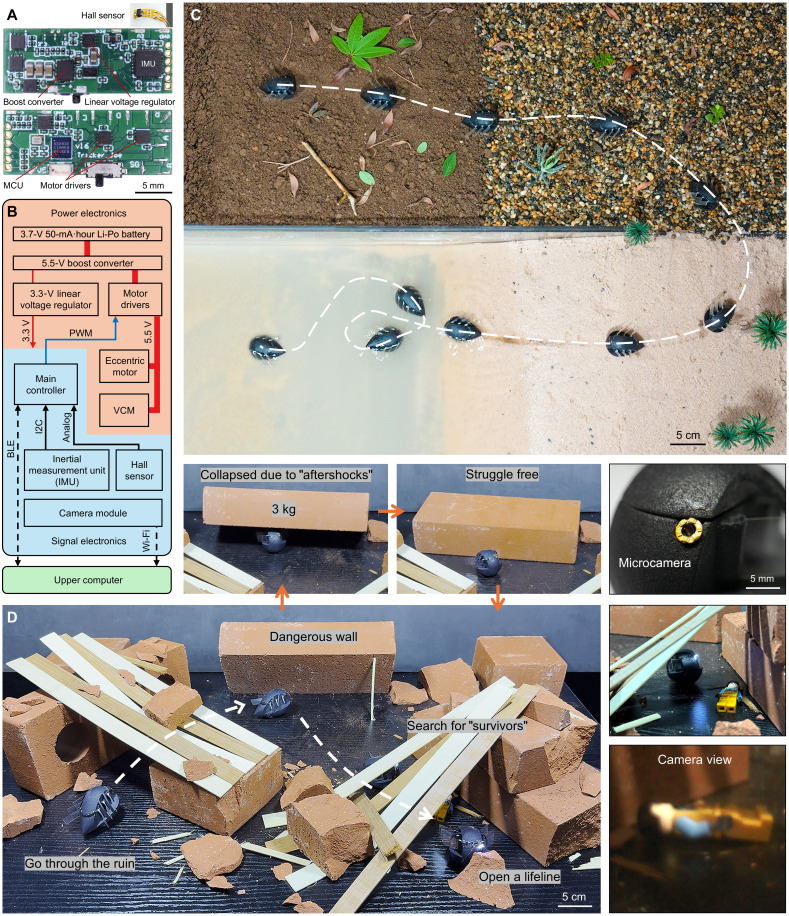
System demonstration. (**A**) Customized main control board and Hall sensor board. (**B**) Simplified schematic of the control system. (**C**) The robot demonstrated locomotion across mud, stones, sand, and water. (**D**) The robot navigated hazards to search for a “survivor.” After a 3-kg wall collapsed because of “aftershocks,” it escaped and continued the wireless search using a microcamera and then cleared the “lifeline” by pushing a brick.

For practical applications, the prototype must be self-contained and support multiple feedback modalities. In this work, a customized miniature main control board (10 mm by 23 mm) and a flexible Hall sensor board (4 mm by 8 mm) were developed in Fig. 6 (A and B) (see Materials and Methods for details). With the help of remote-controlled electronics, the robot demonstrated promising skills, including jumping across mud, stones, and sand and lastly swimming in the water (Fig. 6C). Moreover, in the search mission shown in Fig. 6D, the hard shell protected the robot from external impact, and the robot was able to free itself via jumping. Then, the robot observed a LEGO “survivor” with its microcamera. Last, the robot pushed a 90-g obstacle in small-stroke vibration mode to clear a “lifeline” path.

## DISCUSSION

We demonstrated Leglessbot, an inertia-driven, self-contained, autonomous, centimeter-scale robot with a fully sealed rigid shell. A powerful, mode-adjustable VCM was designed to enable jumping (explosive actuation), full-stroke vibration (medium output and frequency actuation), and small-stroke vibration (high-frequency actuation). The prototype demonstrated promising characteristics and skills on land, including high robustness, the ability to jump out of sand, high-speed movement on sand, load carrying, and continuous jumping. Its passive asymmetric fins convert reciprocating motion into steerable aquatic propulsion. Systematic aquatic motion capture, PIV experiments, and fluid structure interaction CFD simulations were conducted to examine its inertia-driven thrust generation and IDMP. Last, the robot demonstrated amphibious locomotion across various terrains, showing considerable potential for inertia-driven techniques.

The robot’s mechanical and electromagnetic design also plays a critical role in enabling terrestrial locomotion. While small-stroke vibration is the basic motion of conventional VCMs, generating sufficient inertia for jumping is substantially more demanding. A key factor is the ratio between the magnet mass and the total robot mass: Too low a ratio results in insufficient impulse, whereas too high a ratio requires excessive drive power and compromises efficiency. In this study, we selected the magnet-to-body mass ratio to 0.31, balancing output inertia and power consumption under the constraints of a compact electronic system. Moreover, to lengthen the acceleration stroke, we implemented a multicoil cascade design to extend the effective stroke, allowing the magnet to build up momentum over a longer path. This strategy enables the robot to perform impulsive jumping and terrain escape maneuvers. The intermediate regime—full-stroke vibration—offers a balance between frequency and inertial force and proves particularly effective on granular surfaces, where it induces rapid low-amplitude hopping at a higher frequency. The prototype can operate continuously for at least 28 min of jumping at 6.6 Hz, 11 min of small-stroke vibration, and 6 min of full-stroke vibration. The battery life is lower than that of propeller-driven boats because of the lower efficiency of the actuation principle. Future work will study the principle of power efficiency.

Existing swimmers actively turn their propellers, legs (*15*), bodies (*52*, *53*), or fins (*6*, *54*) to move in water. Thus, protecting smaller swimming robots from water pressure generally becomes more complicated and expensive because of limitations in dynamic sealing techniques. In this work, we propose an approach to generating thrust via inertia while reserving the fully sealed rigid shell, which has great potential for miniature deep-sea swimmers.

Frequency-dependent mode adjustment is beneficial for miniature systems but relies on constructed environments. If the vibration source runs within a stable zone, then the prototype will automatically oscillate stably with certain parameters. Then, IDMP is enabled by the different timing of the next stroke, resembling our previous work to a certain extent: eccentric rotation–dependent multidirectional transmission on GASR, a miniature 1.2-g crawling robot (*27*) whose controllable motion depends on the different timing of ground contact. Frequency-dependent multidirectional actuation improves the motion skills of miniature systems and has shown considerable research prospects in this field. Similar vibration-mode adjustments have been reported in recent studies (*55*–*59*). However, stable vibration highly relies on constructed environments (e.g., flat ground and still water). For instance, GASR cannot perform its motion skills on sand. In addition, for Leglessbot in this work, when undergoing incoming flow, the longer fin has higher fluid resistance and affects the motion (see details in note S5 and movie S5).

Future research will focus on improving environmental adaptability by developing feedback control strategies and adaptive structural designs. In addition, optimizing energy efficiency and miniaturization of onboard power systems will be critical for untethered operation in unstructured terrains or underwater environments.

## MATERIALS AND METHODS

### Fabrication

The passive fins were rectangular polyvinyl chloride films cut by a 395-nm laser cutter and were 10 mm wide and 0.05 mm thick. The robot’s shell was fabricated from 3D-printed nylon (PA12, produced by WeNext). The VCM coils were wound with 0.15-mm-diameter enameled copper wire, and the magnets were N52 grade; further details are provided in tables S1 and S2.

### Control system design

To deliver high power (up to 2.5 W) and prevent voltage drops from the 3.7-V and 50-mA·hour battery during actuation, a boost converter (TPS61288, Texas Instruments) was used to supply the power electronics, while a linear voltage regulator (TLV767, Texas Instruments) provided a stable and precise 3.3-V power for the signal electronics. The main controller, based on the BLE SoC (Bluetooth low-energy system-on-chip; nRF52832, Nordic Semiconductor), generates a 10-kHz pulse-width modulation signal to control both the ERM and VCM via motor drivers (DRV8231A, Texas Instruments). Hall sensor (DRV5053, Texas Instruments) signals are acquired using a successive approximation analog-to-digital converter to estimate the VCM magnet’s position through precalibration. An inertial measurement unit (MPU6050, InvenSense) transmits attitude data to the microcontroller unit, providing real-time information on robot orientation (see fig. S3 for details). A first-person-view camera is mounted on the device’s shell, transmitting live video to a host computer via 2.4-GHz Wi-Fi.

### Experimental section

#### VCM force measurement

The force sensor (ME-Meßsysteme, K6D68; accuracy class: 0.2) was used to measure the axial output force generated by the VCM in the three working modes. The force sensor’s sampling frequency is 6000 Hz, which is ~15 times the VCM’s maximum vibration frequency. The VCM was inclined upward at 45° relative to the horizontal plane during measurement, which is the same angle of inclination as the VCM was placed in the robot. Thus, it can be observed that the axial output force curve of the VCM is asymmetric because of the self-weight of the magnet.

#### Robot terrestrial motion measurement

The robot’s terrestrial motion trajectory and jumping trajectory were captured by the motion capture system (VICON, Vantage V5; accuracy: 0.1 mm) at a sampling frequency of 200 Hz. The trajectories were postprocessed using MATLAB to obtain the velocities. The experimental setup of motion measurement is shown in fig. S16.

To measure the robot’s amplitude-frequency-velocity surface, we varied the input voltage and vibration frequency of the VCM using control variables and then calculated the amplitude of the magnet on the basis of the Hall data. When the robot cannot move, it is recorded as unmovable; when the Hall data lack periodicity, indicating that the robot moves irregularly, it is recorded as unstable; and when the robot moved stably, its trajectory was recorded over three repeated trials.

When measuring the robot’s jump trajectory, the robot is powered via the jump wire, and the printed circuit board operates in 15-V output mode. We varied the power of the VCM using pulse-width modulation, from 20% input to 100%, with 20% increments between changes. The jump trajectory was repeated five times each increment and recorded.

The output inertias per cycle of jumping mode and full-stroke vibration mode were calculated on the basis of the initial velocity of the robot, assuming that the magnet remained stationary with the shell. The output inertia in small-stroke vibration mode was obtained by the data from the force sensor above.

#### Robot water motion measurement

A camera (4K; sampling frequency: 60 Hz) was used to look down and record the horizontal plane. The robot is powered via the jump wire. The circuit board selects the 5.5-V output mode, the VCM selects a 60% voltage input, and the amplitude of the magnet is fixed at 3 mm using the Hall sensor. The robot’s vibration frequency was changed in increments of 2.5 Hz, from 20 to 60 Hz, and the trajectory was repeated three times for recording. We used image measurement software (Kinovea) to obtain the robot’s trajectory and processed it in MATLAB to calculate the linear and angular velocities.

#### PIV experiment

The schematic of the PIV experimental setup is shown in fig. S17. Because the fins of the robot are underwater, the high-speed camera (Ispeed-221 mono, Hadland) was placed at the bottom of the transparent tank and recorded from the bottom up at a sampling frequency of 800 Hz. This avoids the effects of refraction from water waves. The horizontal plane at the midpoint of the height of fins in the water was illuminated from four directions with four linear laser lights (power: 100 mW). These laser lights were calibrated so that their fan-shaped beams illuminate the same plane simultaneously. Only the illuminated PIV particles (BtFluid, PSP tracer particles, 50 μm) were recorded.

The image frames are captured as 1392 by 1296 pixels (Px) and processed using MATLAB’s PIVLab (*60*) to calculate the velocity and vorticity fields. In PIVLab, preprocessing is performed first, with high pass and intensity capping enabled to enhance the visibility of particles. Multipass fast Fourier transform window deformation was chosen as the processing algorithm. The interrogation area is 128 Px, the step size is 64 Px, and pass2 (interrogation area, 32 Px; step, 16 Px) and pass3 (interrogation area, 16 Px; step, 8 Px) are also enabled. Correlation robustness chooses the extreme mode. After initially obtaining the velocity field, velocity-based validation was used to remove unreasonable velocity values, leaving 99.8% valid velocities. Although MATLAB can calculate vorticity fields, the velocity fields are still imported into Techplot 360 for vorticity identification using the λci law (fig. S4).

#### Statistical analysis

All data were expressed as the means ± standard deviation. Statistical analysis of the data was performed using OriginPro 2021.

## Supplementary Materials

### The PDF file includes:

Supplementary Notes S1 to S7 Figs. S1 to S17 Tables S1 to S4 Legends for movies S1 to S10 Legend for data S1
