# Supplementary material for: Inertia-driven amphibious robot with asymmetric microundulatory fin arrays
Source: Sci Adv. 2026 Feb 18;12(8):eaea2222. doi: 10.1126/sciadv.aea2222 (PMC12915614; doi:10.1126/sciadv.aea2222)
Supplement: Supplementary file 1 — Supplementary Notes S1 to S7 Figs. S1 to S17 Tables S1 to S4 Legends for movies S1 to S10 Legend for data S1 [file sciadv.aea2222_sm.pdf]

Science Advances
AAAS

Supplementary Materials for

Inertia-driven amphibious robot with asymmetric microundulatory fin arrays

Lingqi Tang *et al.*

Corresponding author: Bing Li, libing.sgs@hit.edu.cn; Bingfu Zhang, zhangbfu@hit.edu.cn; Qiguang He, qiguanghe@cuhk.edu.hk; Hongliang Ren, hren@cuhk.edu.hk; Yao Li, liyao2018@hit.edu.cn

Sci. Adv. **12**, eaea2222 (2026)
DOI: 10.1126/sciadv.aea2222

The PDF file includes:

Supplementary Notes S1 to S7
Figs. S1 to S17
Tables S1 to S4
Legends for movies S1 to S10
Legend for data S1

Other Supplementary Material for this manuscript includes the following:

Movies S1 to S10
Data S1

Note S1. Additional details of the VCM design.

In the design exploration of the VCM, we dealt with the trade-off between VCM weight, size, output, and actuation power. For example, increasing the number of windings or the actuation voltage can enhance the magnet velocity, but this inevitably increases the overall system weight. The final parameters are shown in Fig. S1 and Tables S1 to S4. Three coils are fixed to the VCM shell, and the magnet slides into the inner space. The fit clearance is designed to be 0.25 mm. The magnet assembly consists of two individual magnets bonded together using a DP420 epoxy adhesive (3M). Coil 1 and coil 3 are the same, while coil 2 is longer than the others. The longer coil design fits the increased magnet length due to the bonding structure. Finally, our previous design explorations indicate that the inertial mass ratio of the magnet should not fall below 0.3 to enable effective jumping.

The three coils are connected as shown in Fig. S1B. Coil 1 and Coil 3 are connected in series to form Coil A, which is then connected in parallel with Coil 2. This connection increases the current of coil 2 and improves efficiency because coil 2 always provides actuation force, unlike the other two coils. The overall VCM is actuated by one channel of the motor driver. We chose the 5.5 V power supply and 10 kHz PWM to actuate the VCM. The static stalling current of the VCM is obtained as 0.72 A. The following calculation verifies the VCM power in different modes.

For the series configuration of Coil 1 and Coil 3, the resulting combination is referred to as Coil A. The equivalent inductance and resistance of Coil A are the sum of the respective parameters of Coil 1 and Coil 3.

$$L_A = L_1 + L_3$$

$$R_A = R_1 + R_3$$

The impedance of Coil A is then determined based on its total resistance and inductive reactance.

$$Z_A = R_A + j\omega L_A$$

In the next stage, Coil A is connected in parallel with Coil 2. The overall impedance of the system is thus calculated as the parallel combination of the impedance of Coil A and Coil 2. By substituting the measured parameter values into the expressions, the total series impedance is obtained:

$$Z_2 = R_2 + j\omega L_2$$

$$Z_{\text{total}} = \frac{Z_A \cdot Z_2}{Z_A + Z_2}$$

$$Z_{\text{total}} = 6.15 + j40.64\ \Omega$$

$$|Z_{\text{total}}| = 41.10\ \Omega$$

$$\angle Z_{\text{total}} = 81.39°$$

The resulting total impedance is:

$$Z_{\text{total,series}} = Z_{\text{VCM}} + R_{\text{Drv}} = (6.15 + j40.64) + 1.5 = 7.65 + j40.64\ \Omega$$

$$\left|Z_{\text{total,series}}\right| = \sqrt{7.65^2 + 40.64^2} \approx 41.36\ \Omega$$

For a square-wave driving signal with a peak amplitude of 5.5 V and a 50% duty cycle applied to an inductive load, the effective RMS voltage is calculated accordingly:

$$V_{\text{RMS}} = V_{\text{peak}} \cdot \sqrt{D} = 5.5 \cdot \sqrt{0.5} \approx 3.89\ \text{V}$$

$$I_{\text{RMS}} = \frac{V_{\text{RMS}}}{|Z|}$$

Under this condition, the RMS current is approximately 94 mA.

In the jump actuation mode, where the VCM is driven by a full-power symmetric bipolar square wave of ±5.5 V at a switching frequency of approximately 83.3 Hz (according to the actual jumping time of 18 ms), the impedance is recalculated for the lower frequency:

$$Z = R + j\omega L = 7.65 + j\omega \cdot 0.001902798$$

The resulting RMS current under this condition is 713 mA. Although the battery can supply this level of peak current, the VCM requires a cooling period (>100 ms) to prevent overheating. Consequently, the maximum achievable full-power jumping frequency is limited to approximately 6.6 Hz.

To ensure jumping capability, the ratio between the magnet mass and total robot mass should exceed 0.3. As shown in Table S4, certain components—such as the PCB, battery, and ERM motor—exhibit sharply degraded performance when their sizes are scaled below this range. In addition, both the VCM shell and robot shell must maintain sufficient strength to withstand impact. Under these constraints, miniature components were selected and designed, and the magnet mass was fixed at 7.4 g (≈ 0.31 of the total weight). The coils were subsequently designed to match the magnet parameters and provide sufficient actuation power without exceeding the battery's current and power constraints.

Note S2. **Additional details of COMSOL simulation setup and MATLAB post-processing.**

The model and governing equations:

The 2D fluid-solid coupling model was selected in COMSOL and the added physical field interfaces were laminar flow (fluid domain), solid mechanics (solid domain), and fluid-solid coupling (multiphysics field). The governing equations for the fluid section are the Navier-Stokes equations:

$$\rho_f \frac{\partial \mathbf{u}}{\partial t} + \rho_f(\mathbf{u} \cdot \nabla)\mathbf{u} = \nabla \cdot [-p\mathbf{I} + \mu(\nabla \mathbf{u} + (\nabla \mathbf{u})^T)] + \mathbf{F}_f$$

$$\nabla \cdot \mathbf{u} = \mathbf{0}$$

Where: ρ_f is the fluid density, **u** is the fluid velocity field, p is the pressure, μ is the dynamic viscosity, $\mathbf{F}_f$ is the volumetric force acting on the fluid, and **I** is the identity tensor.

In the solid mechanics section, because of the large deformations observed in the fin, the set of elastodynamics equations applicable to large deformations was chosen:

$$\rho_s \frac{\partial^2 \mathbf{d}}{\partial t^2} = \nabla \cdot \mathbf{P}^T + \mathbf{F}_s$$

This equation describes the motion of a solid under the action of inertial forces, stress, and volumetric forces. Here, ρ_s is the solid material density, **d** is the displacement field of the solid, **P** is the first Piola-Kirchhoff stress tensor, and $\mathbf{F}_s$ is the volumetric force acting on the solid.

The first Piola-Kirchhoff stress tensor **P** is related to the second Piola-Kirchhoff stress tensor **S** through the deformation gradient tensor **F**:

$$\mathbf{P} = \mathbf{FS}$$

The deformation gradient tensor **F**, which characterizes the local geometric changes during deformation, is defined as

$$\mathbf{F} = \mathbf{I} + \nabla \mathbf{d}$$

where **I** is the identity tensor. The determinant of the deformation gradient,

$$J_F = \det(\mathbf{F})$$

represents the local volume change.

The second Piola-Kirchhoff stress tensor **S** is related to the Green-Lagrange strain tensor **ε** by a linear elastic constitutive relation:

$$\mathbf{S} = \mathbf{C} : \boldsymbol{\varepsilon}$$

where the Green-Lagrange strain tensor is given by:

$$\boldsymbol{\varepsilon} = \frac{1}{2}(\mathbf{F}^T\mathbf{F} - \mathbf{I})$$

and **C** is a fourth-order elastic stiffness tensor. For an isotropic linear elastic material, **C** depends on Young's modulus E and Poisson's ratio ν, which can be written compactly as:

$$\mathbf{C} = \mathbf{C}(E, \upsilon)$$

Whether large deformation is turned on or not is automatically determined by the software.

The fluid–solid coupling was configured as fully coupled, with the dynamic mesh enabled. The fluid pressure was applied to the solid as a boundary load, and the solid displacement induced deformation of the fluid domain, thereby affecting the fluid solution.

Mesh construction:

The fluid region was divided using a quadrilateral mesh and encrypted near the wall. For the solid domain, the fin was uniformly divided into 20 elements, which provided sufficient accuracy while avoiding excessively fine meshing. This improves computational efficiency and also helps with post-processing the results.

Parameters setup:

The default parameters of liquid water were used for the fluid. For the fin material, the density $\rho_s = 1380\ \mathrm{kg/m^3}$, $E = 2500$ MPa, $\upsilon = 0.35$ were adopted. The initial velocity and pressure fields were set to zero, and the outer boundary of the flow domain was specified as a no-slip wall.

The simulation primarily focused on the fluid–fin interaction; therefore, the collision process of the VCM was not included. Instead, the VCM collision was simplified as a square-wave displacement input. Based on the robot's observed motion in experiments, the amplitude of the square-wave displacement was set to 1.1 mm. This displacement was applied to one end of the fin via a displacement constraint. The displacement direction was set to form a 45° angle with the fin's longitudinal axis, consistent with the fin installation on the robot. Transient simulations were performed using an implicit solver with a relative tolerance of 0.001.

Result output in COMSOL:

After completing the simulation in COMSOL, the pressure field on both sides of each of the 20 mini-fins is obtained at every time point. The position of each mini-fin at each time point is also extracted and used to reconstruct the fin shape.

Post-processing in MATLAB:

In MATLAB, the pressure is line-integrated on each side of the 20 mini-fins, and the difference between the two sides is computed to obtain the net thrust of each individual mini-fin. In both experiments and simulations, we observed substantial fin bending; therefore, the thrust direction (angle) of each mini-fin is computed individually based on the reconstructed fin shape to ensure accuracy. For each mini-fin, the thrust angle and the lever arm—defined as the distance from the robot's center of mass (which coincides with the center of rotation) to the midpoint of the mini-fin—are calculated. The total thrust and torque generated by the entire fin are then obtained by summing the contributions of all mini-fins according to their positions and lever arms. Finally, time integration yields the linear momentum and angular momentum.

Note S3. Effect of fin length on robotic performance: inference and experimental validation

In the original manuscript, we inferred the effect of fin length based on the experimental results in Figs. 3F and 3G. These results showed that fin length primarily shifts the resonance frequency while having only a minor influence on the peak magnitude at resonance. Under this assumption, it was reasonable to hypothesize that shortening the right fin (from 17.5 mm to 15.0 mm) would increase the resonance-frequency difference between the two sides (Fig. S9A), whereas extending it (from 17.5 mm to 19.0 mm) would reduce this difference (Fig. S9B). The corresponding time-averaged net torque ($\overline{\tau_N}$)-frequency curves are shown in Fig. S9C. This inference suggested that increasing the fin-length difference should enhance variations in $\overline{\tau_N}$ and thus increase the robot's angular velocity, whereas decreasing the difference should facilitate straighter forward motion at the cost of reduced turning ability.

To rigorously validate this inference, we performed additional experiments using fins of different lengths, as detailed in Fig. S10. As shown by the comparison in Fig. S10C, the L20-R19 combination exhibits a generally smaller angular velocity than L20-R17.5. Conversely, the L20-R16 combination produces markedly higher angular velocity than L20-R17.5 across most frequencies, reaching up to 35°/s at 47.5 Hz. These comparison results confirm the primary conclusion: the dominant factor governing angular velocity is the resonance-frequency difference introduced by the fin-length asymmetry. At the same time, the experiments also reveal secondary effects not fully captured in the initial inference, such as mild waveform scaling and vertical shifts. These effects arise because changing fin length simultaneously alters the hydrodynamic contact area and proportionally modifies the inter-mode spacing among all resonance modes. Nonetheless, these secondary effects do not change the dominant role of resonance-frequency difference.

Overall, the combined inference and experimental results demonstrate that selecting an appropriate fin-length difference allows a trade-off between high angular velocity and controllable straight-line motion.

Note S4. Effect of fin thickness on robotic performance.

The fin thickness affects resonant frequency. According to ANSYS Workbench simulations, the wet modal resonance frequency of the fin is proportional to its thickness. Therefore, the $\bar{\tau}$-f curve for fins of other thickness is expected to resemble a proportionally stretched or compressed version of the curve for the 0.05 mm thick fins.

Experiments were conducted using 0.025 mm and 0.075 mm thick fins, and their performance is compared with the case of 0.05 mm thickness. The results are presented in Fig. S11. Using 0.05 mm thick fins, the robot has three frequencies that yield straight motion (Fig. 3B). With 0.025 mm thick fins, it exhibited four; and with 0.075 mm thick fins, it showed two, indicating a clear scaling trend. The curves do not exhibit full proportional similarity to those of the 0.05 mm thick fins. This discrepancy may occur because the scaling effect causes different resonance orders—depending on the fin thickness—within actuator's frequency range (20 – 60 Hz). Although the resonance orders used for each thickness may differ, this does not affect IDMP.

Thickness also affects the fluid accumulation effect shown in Fig. 4E(b) and Fig. 4H. The robot moves by accumulating the thrust difference generated during the forward and backward stroke processes. If the fins become thicker, their stiffness increases, resulting in reduced deformation during stroke. This diminishes the fluid accumulation effect, thereby slowing the robot's speed. The ultimate case is rigid fins. Conversely, if the fins become thinner, their elasticity decreases, causing them to yield to water flow and hard to rebound. This also reduces the fluid accumulation effect and slows the robot's speed. The ultimate case is with no fins. Therefore, the optimal thickness should deal with the trade-off between stroke and rebound difficulty.

Note S5. The effect of water flow on robotic performance.

Flowing water influences the movement of the robot, as shown in Fig. S12 and movie S5. When subjected to flow from the front, the longer fins (left side) experience higher fluid resistance. Consequently, the robot achieved dynamic equilibrium with the oncoming flow at 33 Hz, corresponding to a right turn in still water. The body yaw angle also tilted ~30°. The flow velocity measured at the robot's location is ~45 mm/s.

The current motion control of the robot operates in an open-loop configuration. Therefore, if disturbances disrupt the dynamic equilibrium, the robot turns away (Movie S5).

Note S6. The effect of surface properties on robotic performance.

Surface properties affect the robot's motion, as the vibrational actuation relies on repeated contact and interactions between the robot’s base and the underlying surface. Based on the EVA plate under small-stroke vibration, comparative experiments were conducted on sponge (softer) and aluminum (harder) plates, as shown in fig. S13.

As surface stiffness increases, the maximum stable frequency decreases. On harder surfaces, higher momentum leads to stronger ground impact upon landing, resulting in bouncing and unstable motion. In contrast, softer surfaces can absorb part of the impact energy, allowing stable motion at higher momentum. However, this also implies that increasing the frequency on the sponge surface from 30 Hz to 60 Hz does not yield a substantial improvement in speed. As a result, linear velocity is generally higher on harder surfaces. To be noted, the small-stroke vibration mode is not designed for sandy lands, as it may sink into the sand.

Note S7. Process of data acquisition, processing and visualization of Fig. 2D

All data in Fig. 2D were directly derived from experimental measurements. For the VCM setup, the input parameters are the drive voltage and the Hall threshold (desired vibration amplitude). The Hall threshold is set above the base value. When the Hall sensor detects a magnetic field exceeding this threshold, the coil swaps its current direction. The output data consists of: (1) the robot's motion trajectory and velocity captured by the VICON motion capture system, and (2) the Hall sensor feedback curve (Fig. S15A).

By varying the drive voltage and Hall threshold at predetermined intervals, the table of relationships between input parameters and output data (Fig. S15B) was obtained. This table was visualized using OriginPro 2021, leading to the representation in Fig. 2D, which has multiple vertices and straight boundaries.

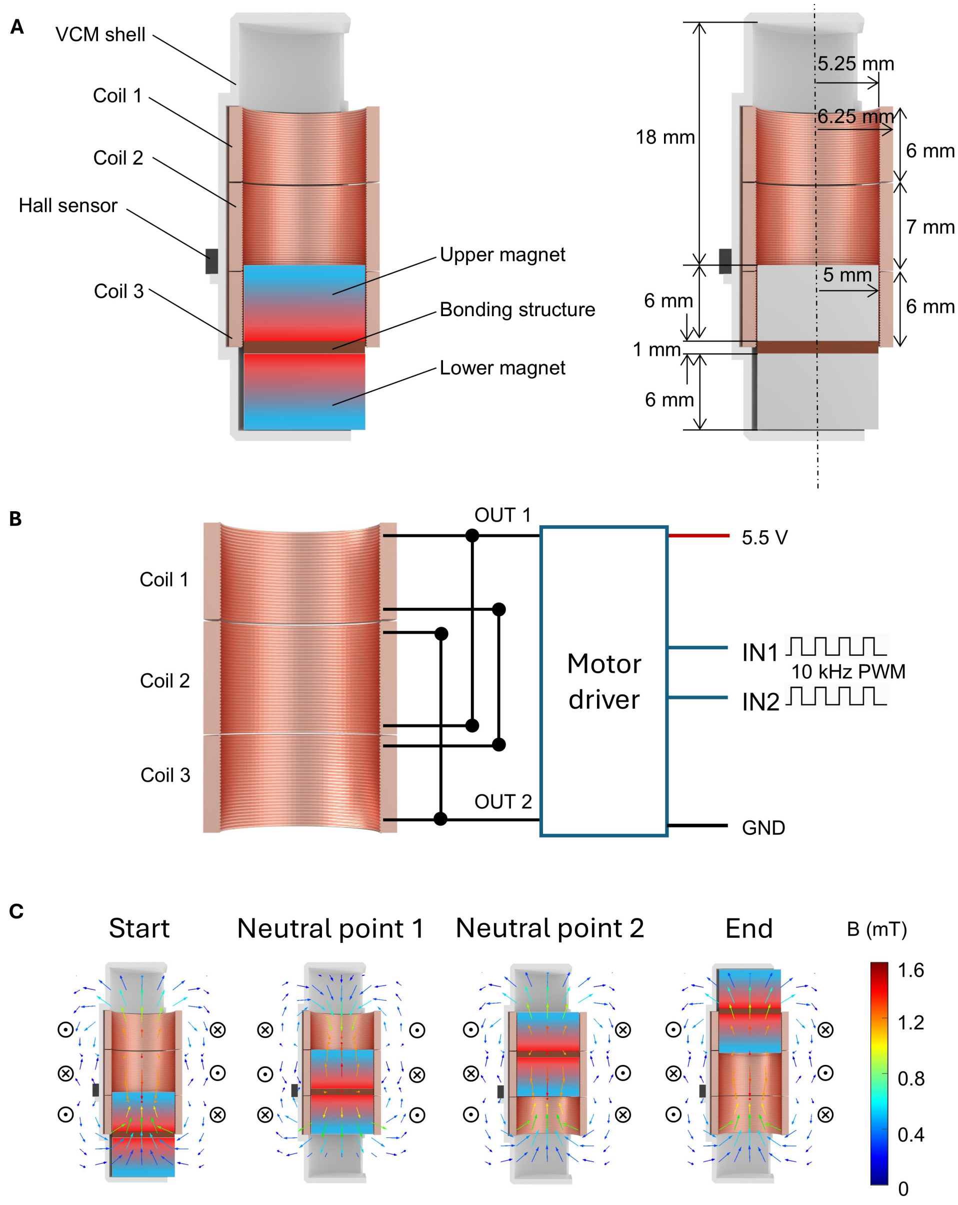

Fig. S1. VCM design and actuation. (**A**) 3D model of the VCM. The overall magnet consists of two magnets with head-to-head bonded. (**B**) Coil 1 and Coil 3 connect in series and then connect with Coil 2 in parallel. This design gives Coil 2 a larger current than the other two coils. (**C**) Schematic diagram of the full stroke process with magnetic field details and current direction.

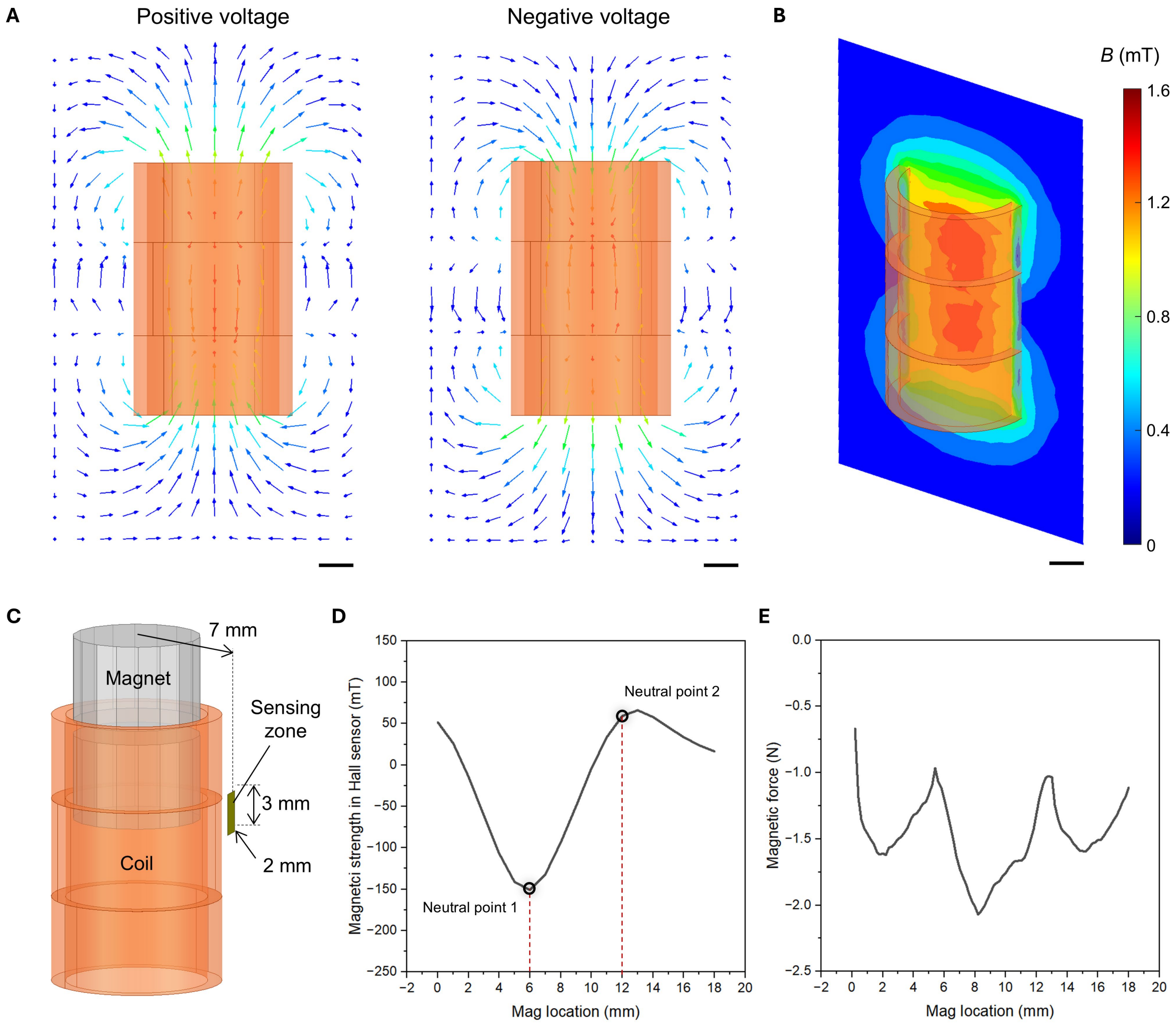

Fig.S2. Electromagnet simulation. (**A**) Magnetic Induction vector produced by the coils with positive and negative voltages, respectively. (**B**) Magnetic field strength produced by the coils; the maximum strength was 1.6 mT. (**C**) Comparison of the location and size of the Hall sensor with the coils. (**D**) Simulated average strength perpendicular to the hall sensor during the whole stroke of the magnet. (**E**) The simulated force acting on the magnet varies with its location in jumping motion at 15 V. When the magnet approaches the two neutral points, the coil voltage toggles to keep pushing the magnet. The output force revealed the output inertia of 26.53 mN·s, which was close to the experimental result (20.2 mN·s). Scale bars, 2.5 mm.

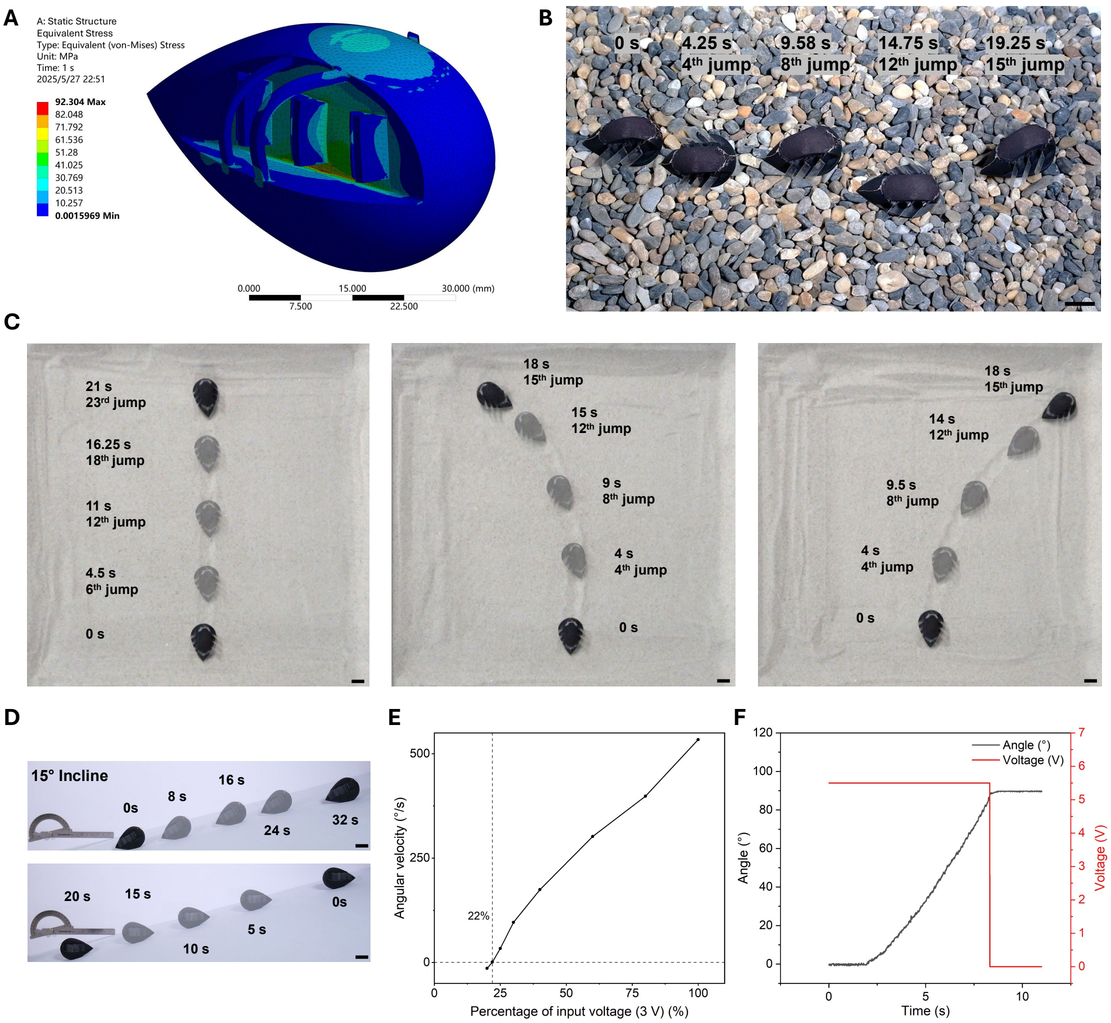

Fig. S3. Additional details of the terrestrial tests. (**A**) Simulation result of the shell under pressure on the top surface (227 mm^2). The material is nylon 66. The yielding load is 347 kg, and the ultimate strength is 498 kg. (**B**) The robot jumped through stones. The average length of the stone particles is 14 mm (~0.24 BL). (**C**) The robot jumped through soft sand. The jumping turning motion was enabled by ERM actuation (20 ms) at the landing timing. After 15 jumps, the robot turned ~60 degrees. (**D**) the robot working in small stroke vibration mode can move on a 15-degree incline and can reach speeds of ~11.2 mm/s (~0.2 BL) and ~22.7 mm/s (~0.4 BL) up and down the incline, respectively. (**E**) Angular velocities on flat ground vary with the input voltage of the ERM. Notably, at voltages below 0.66V (3V×22%), the robot changes its steering direction. (**F**) The robot controlled its yaw angle from 0 to 90° actuated by the ERM with the feedback of the IMU, when the angle reached 86°, the ERM stopped to let the robot slide to the target angle. The controlled error is less than 2°. Scale bars, 20 mm.

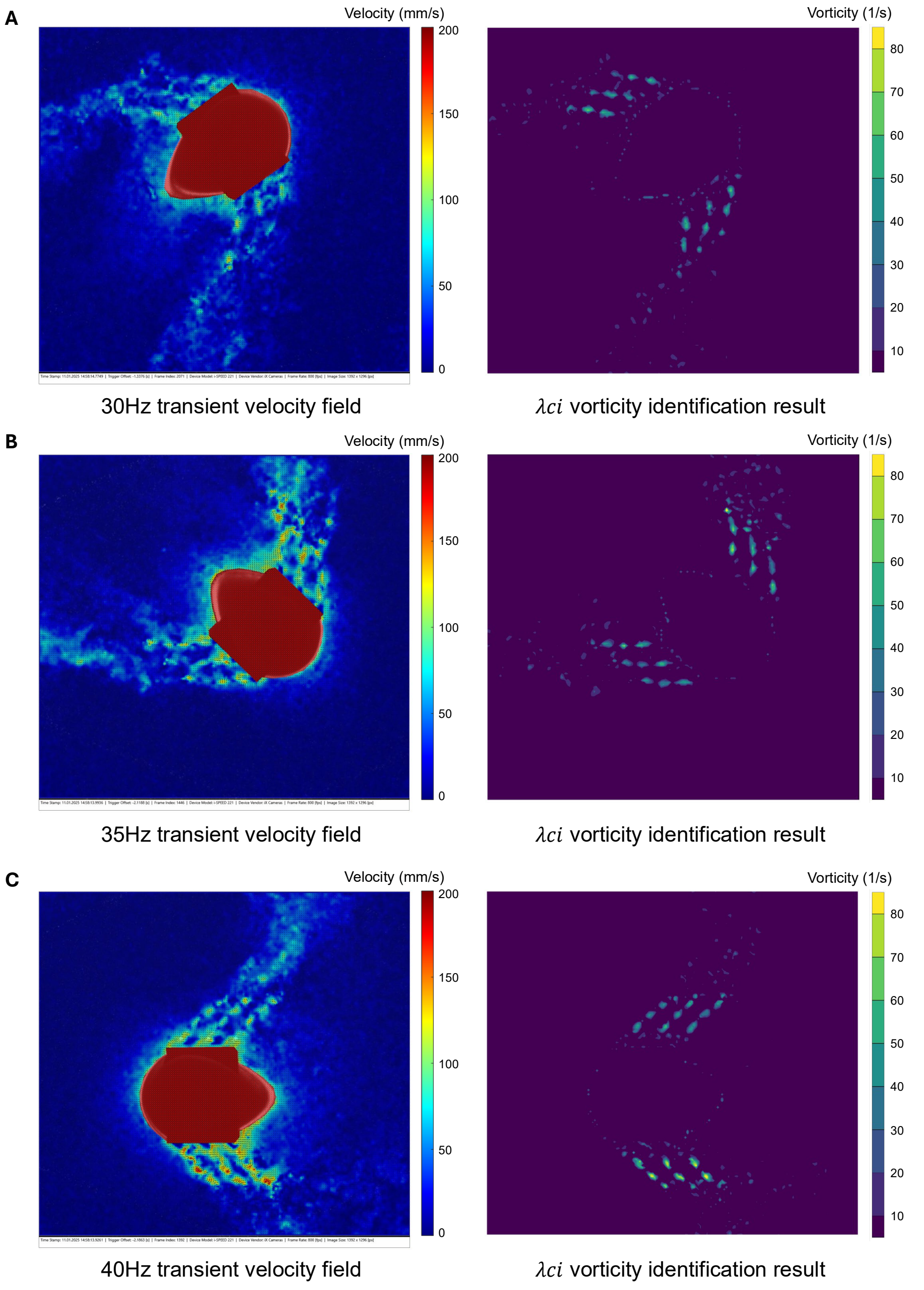

Fig. S4. Results of vorticity identification using λci law. (**A-C**) Transient velocity fields (processed by PIVLab at three typical frequencies (30, 35, and 40 Hz) and corresponding vorticity (processed by Techplot 360) identification results. The results show that the robot generated a regular arrangement of strong vortices (composed of separate annular flows) during vibration and these pseudovortices are transmitted outward with fin fluctuations. The λci law is mainly used to accurately identify the location and intensity (size) of vortices. It is a non-negative scalar and does not contain directional information.

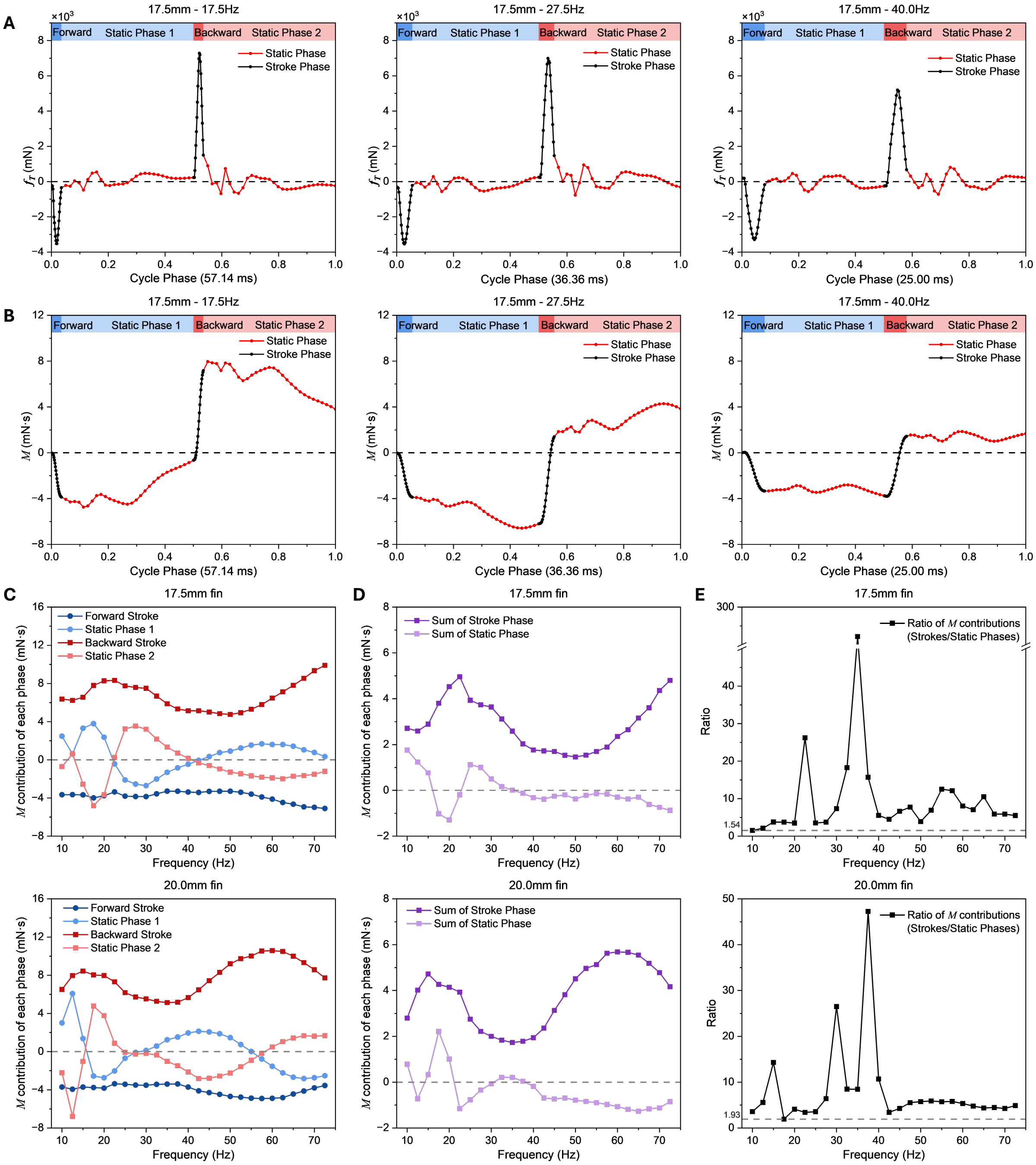

Fig. S5. Thrust and momentum generation processes at each frequency for 17.5 mm and 20 mm fins. (**A**) Transient thrust-time curves of the 17.5 mm fin at three typical frequencies (17.5, 27.5, and 40.0 Hz). (**B**) Momentum thrust-time curves of the 17.5 mm fin at three typical frequencies (17.5, 27.5, and 40.0 Hz). Curves of the 20.0 mm fin and other frequencies are basically similar and summarized in fig. S5C-S5E. (**C**) Summary curves of the momentum contributions of the four phases in one vibration cycle. (**D**) Summary curves of the momentum contributions of the stroke and static phases in one vibration cycle. (**E**) Momentum contribution ratio of stroke and static phases. The curves show that momentum is mainly generated by the stroke process at all frequencies.

A

17.5 mm fin

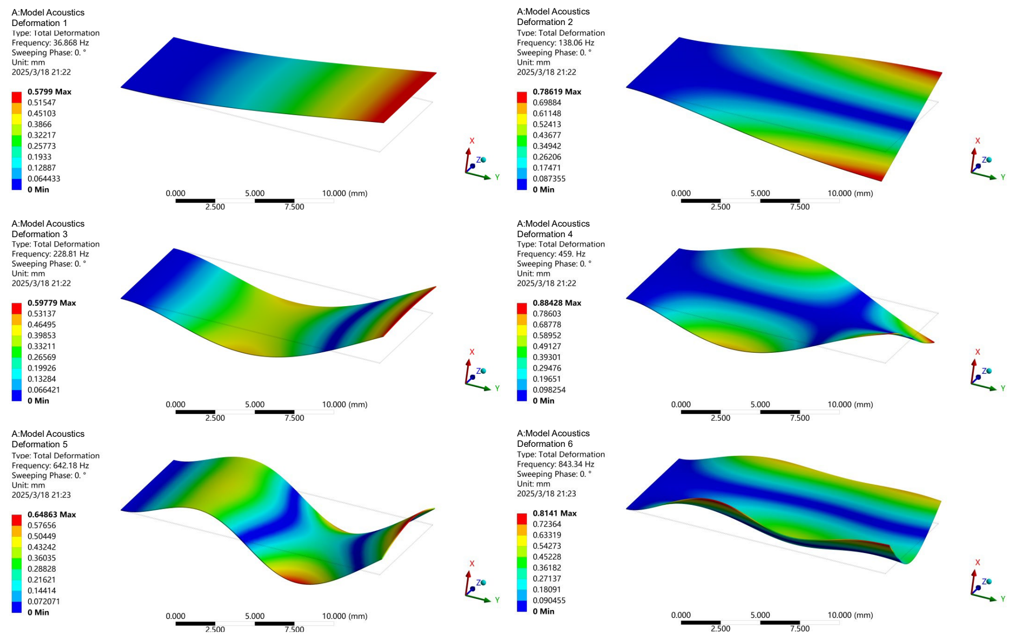

B

20.0 mm fin

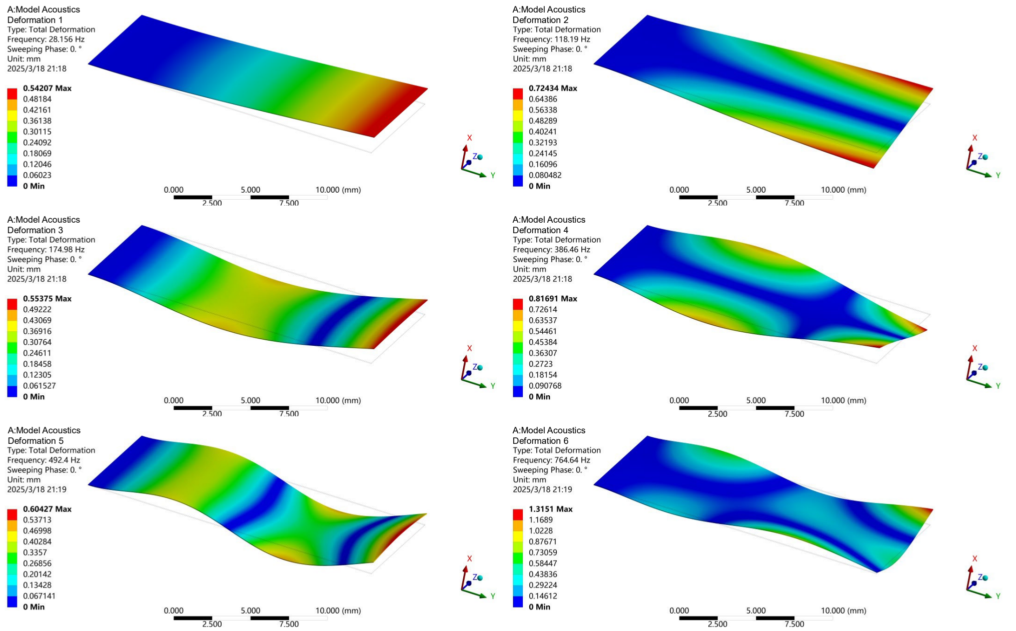

Fig. S6. Wet modal simulation results in Ansys Workbench 2022 R2. (**A**)(**B**) The wet mode resonance frequencies of all orders for the 17.5 mm and 20 mm fin, respectively. The results show that the wet mode resonance frequencies of all orders for the 20 mm fin lag behind those of the 17.5 mm fin. Although the first and second wet mode resonance frequencies of the fin differ from the results in Fig. 3G, the relative relationships are correct. The difference may be caused by the direction of vibration of the fin inclined 45° to the direction of motion.

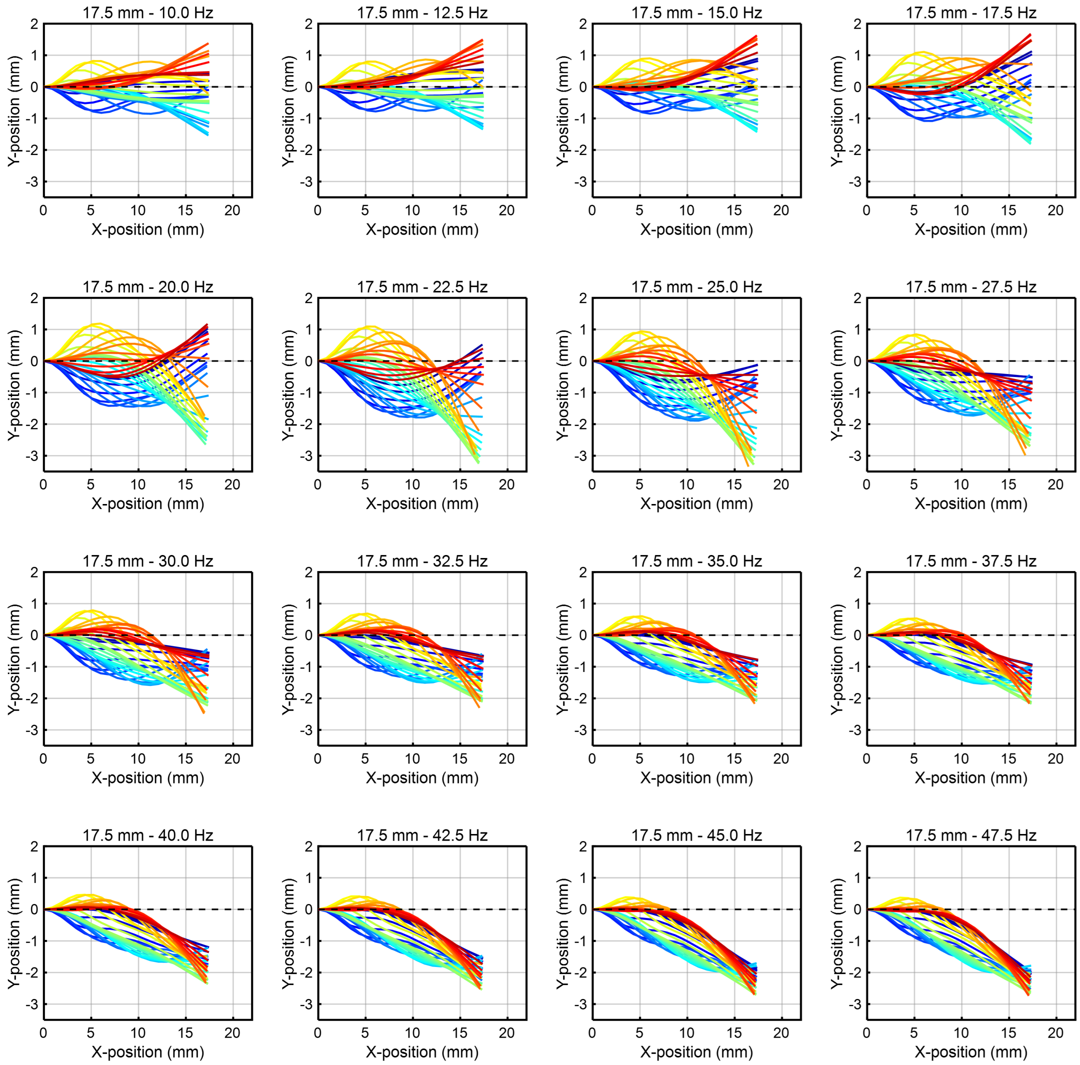

Fig. S7. Vibration profiles of a 17.5 mm fin at each frequency.

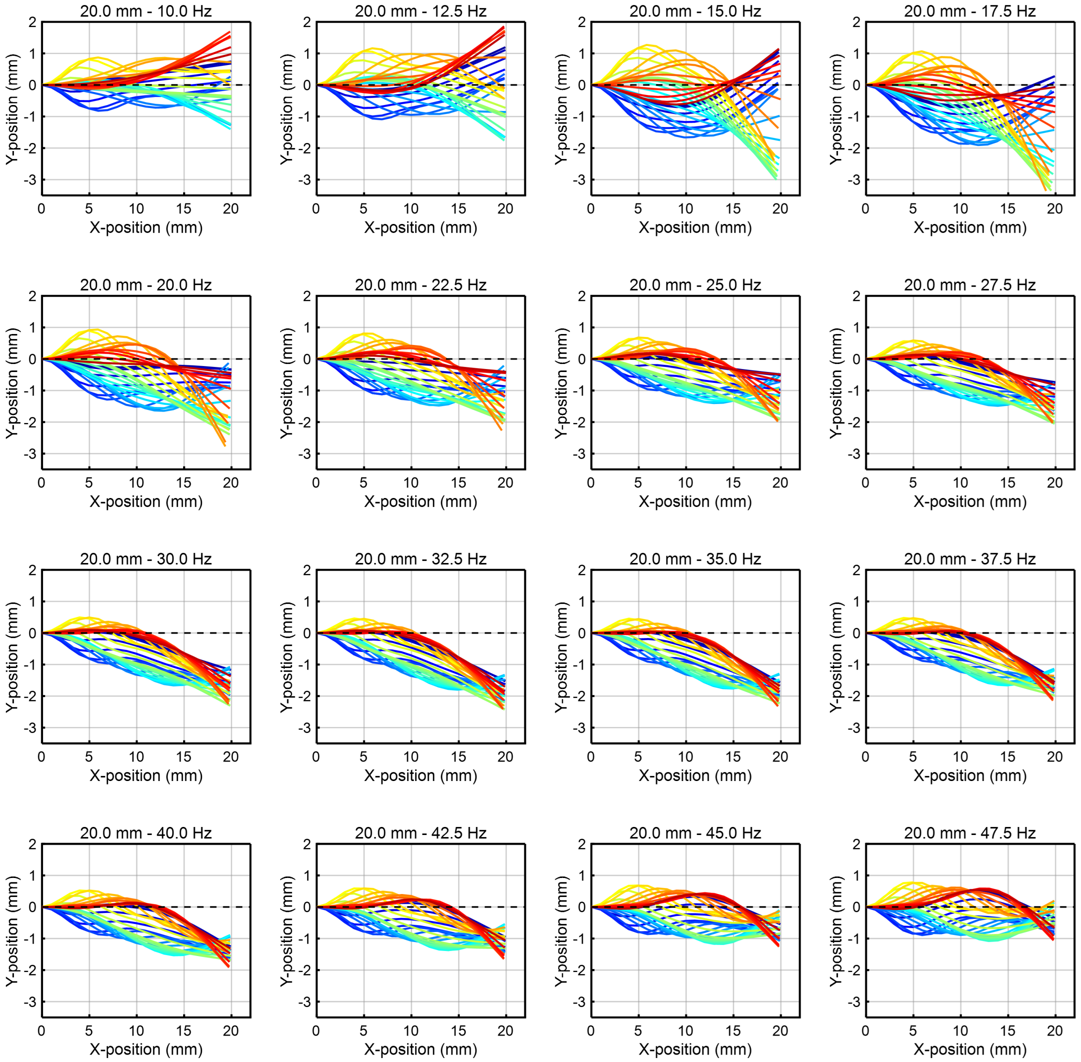

Fig. S8. Vibration profiles of a 20.0 mm fin at each frequency. The comparative results between Fig. S7 and Fig. S8 indicate that the vibration patterns of the 20 mm fin generally lagged behind those of the 17.5 mm fin.

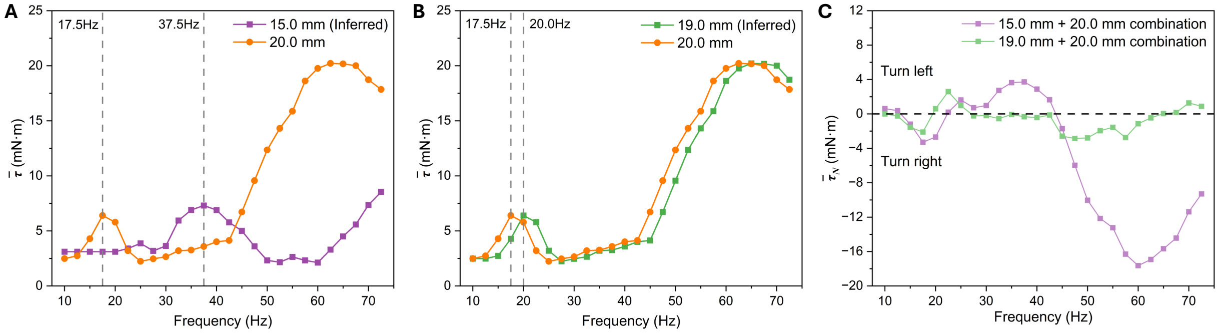

Fig. S9. Schematic diagram of the inferred effect of fin length. (**A**) The inferred 15.0 mm curve is obtained by translating the 17.5 mm curve to the right. (**B**) The inferred 19.0 mm curve is obtained by translating the 20.0 mm curve to the right. (**C**) Time-averaged net torque under inference conditions.

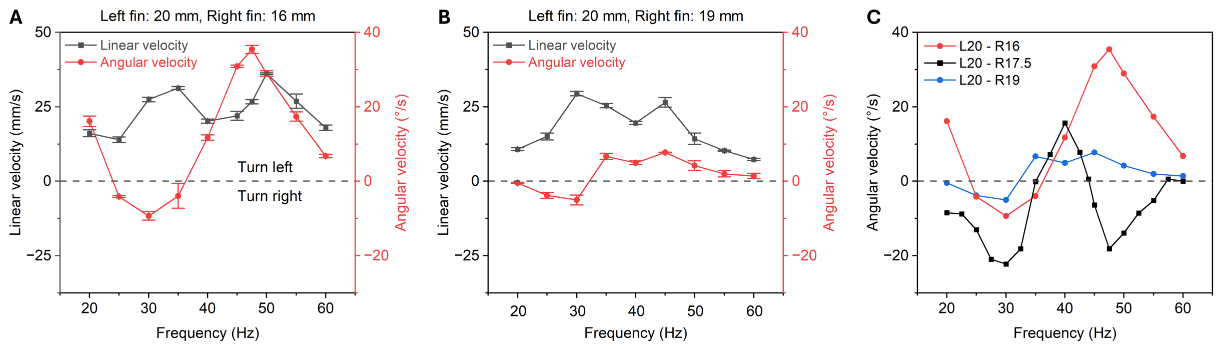

Fig. S10. Aquatic test results with different fin lengths. The measured aquatic linear and angular velocity curves versus frequency after (**A**) increasing and (**B**) decreasing the length difference. All thickness is 0.05mm. Error bars: standard deviation for $n = 3$ samples. (**C**) The average angular velocity comparison for three combinations of fin length differences.

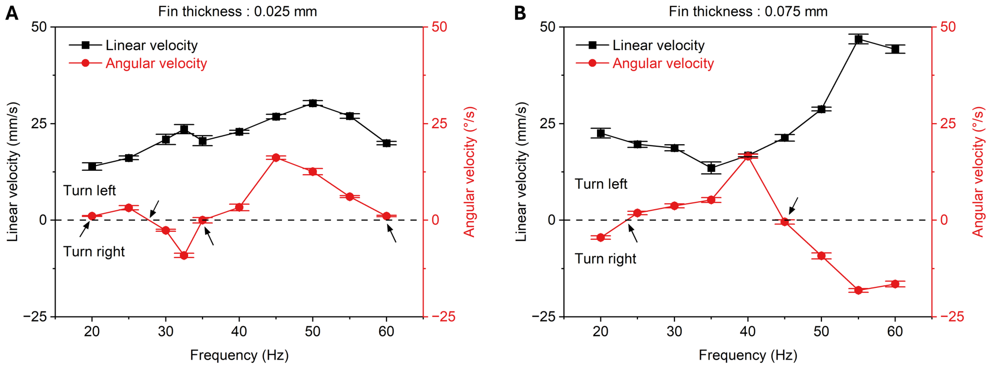

Fig. S11. Aquatic test results with different fin thickness. The measured aquatic linear and angular velocity curves versus frequency of (**A**) 0.025 mm and (**B**) 0.075 mm thick fins. Error bars: standard deviation for $n = 3$ samples. In this test, the fin-length configuration was kept constant, with 20.0 mm and 17.5 mm on the left and right sides, respectively.

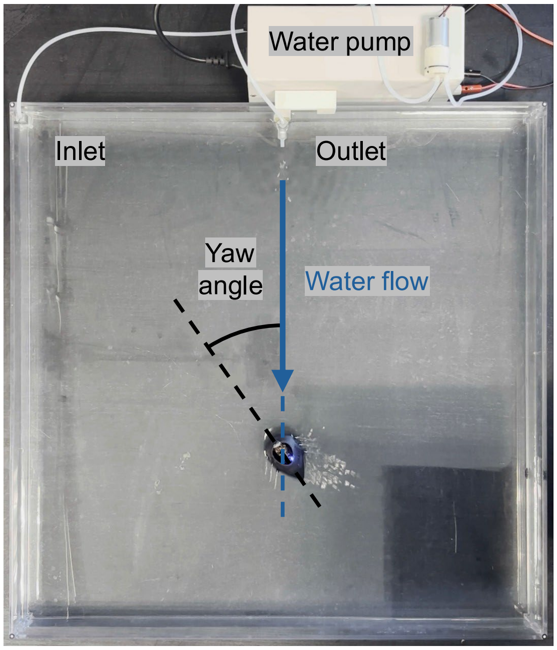

Fig. S12. Water flow test. The inlet at the bottom of the tank did not affect the robot's movement.

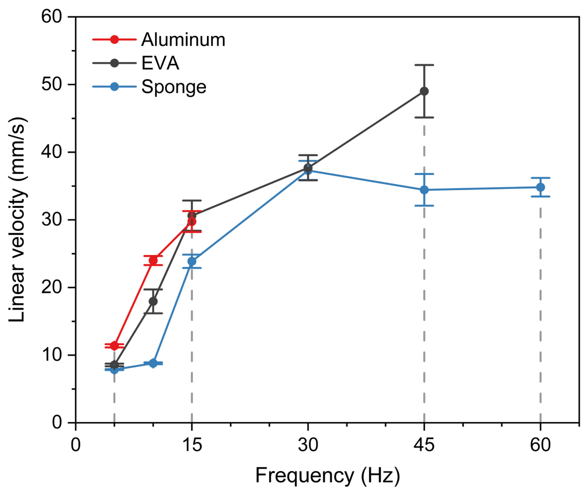

Fig. S13. Stable motion velocity vs frequency on different surfaces. Under a fixed input voltage and amplitude of the VCM, the frequency was varied. The robot's trajectory was classified as stable if it remained within a 15 cm-long and 5 cm-wide strip, and only stable motion results were plot. Error bars: standard deviation for $n = 3$ samples.

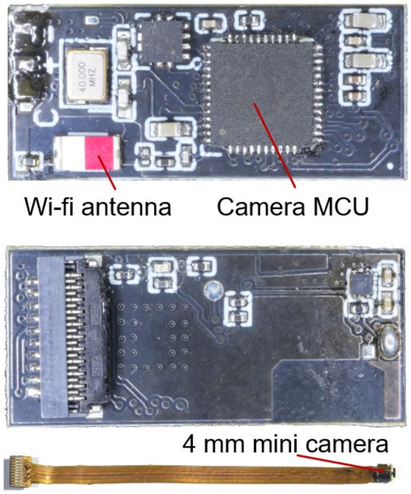

Fig. S14. PCB of the camera module. The camera MCU is TXW817 from TaiXin semiconductor. The MCU reads the camera data via UVC port and transfers the camera data to the upper computer via 2.4 G Wi-Fi.

A

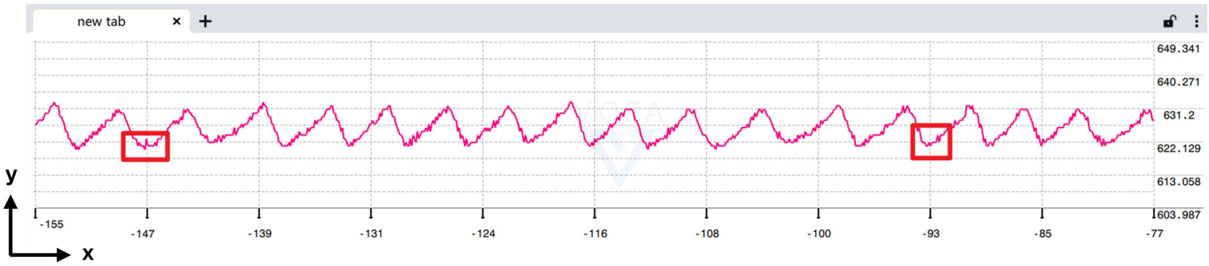

B

% / HT	42	45	48	50	51	54	57	60	63	65
Base	F (Hz)<br>HA (count)<br>V (mm/s)	UM	UM	UM	UM	UM	UM	UM	UM	UM
1	UM	UM	93.46<br>3<br>4.3169	97.56<br>4<br>5.6387	100<br>4<br>5.8189	104.16<br>4<br>8.0083	US	US	US	US
5	UM	UM	44.64<br>7<br>5.5389	55.56<br>8<br>7.4688	61.54<br>8<br>10.6483	70.42<br>8<br>12.536	83.68<br>8<br>14.7525	82.64<br>10<br>19.93	82.3<br>12<br>25.465	84.38<br>13<br>28.1364
10	UM	20.2<br>11<br>2.1852	31.74<br>11<br>6.5728	38.84<br>12<br>9.5788	43.86<br>12<br>11.6446	49.62<br>12<br>15.5943	54.5<br>14<br>19.5599	62.5<br>14<br>24.0416	US	US
15	UM	17.4<br>15<br>3.4405	23.8<br>17<br>8.7971	30.76<br>17<br>13.7089	36.36<br>17<br>15.3323	43.48<br>18<br>21.48456	49.14<br>17<br>23.3898	US	US	US
20	UM	16.08<br>19<br>4.2908	22.84<br>20<br>10.736	28.5<br>20<br>15.2791	29.2<br>21<br>17.2271	33.62<br>21<br>24.5361	US	US	US	US
25	UM	16.74<br>23<br>5.3508	19.42<br>24<br>12.5443	27.4<br>24<br>17.6211	28.58<br>24<br>22.0637	US	US	US	US	US
30	UM	UM	US	US	US	US	US	US	US	US

US: Unstable; UM: Unmovable; %: % of 5.5 V; HT: Hall threshold; F: Frequency; HA: Hall Amplitude; V: Velocity

Fig. S15. Intermedia from of the data in Fig. 2D. (**A**) Hall sensor feedback curve. Calibrations indicate: On the y-axis, 6.6 Hall amplitude counts = 1 mm physical amplitude; On the x-axis, 1 data point = 5 ms. Ten or more cycles are selected to calculate the average frequency and amplitude. (**B**) Table of relationships between input parameters and output data. Each frequency, Hall amplitude and velocity is the average of three samples.

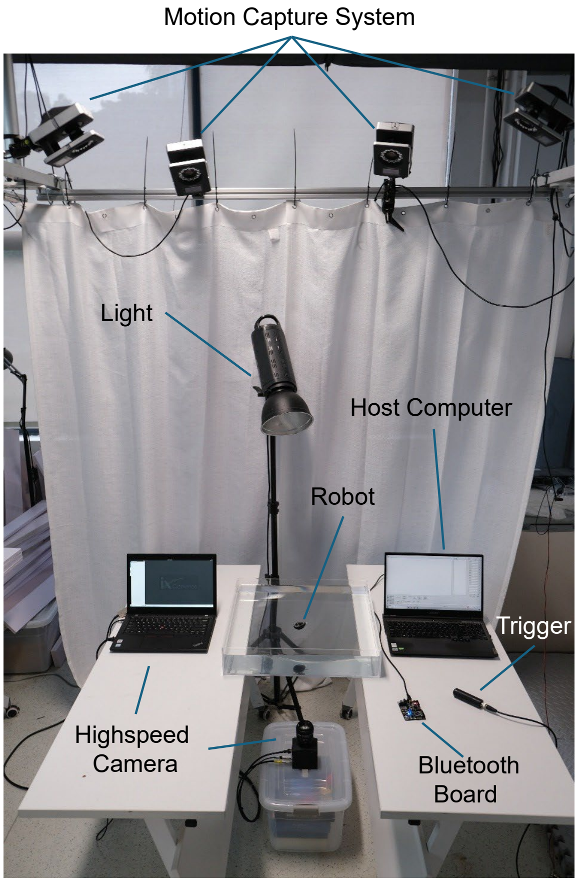

Fig. S16. Testing setup 1: High-speed recording experimental site. The same site was also used for other experiments on other terrains.

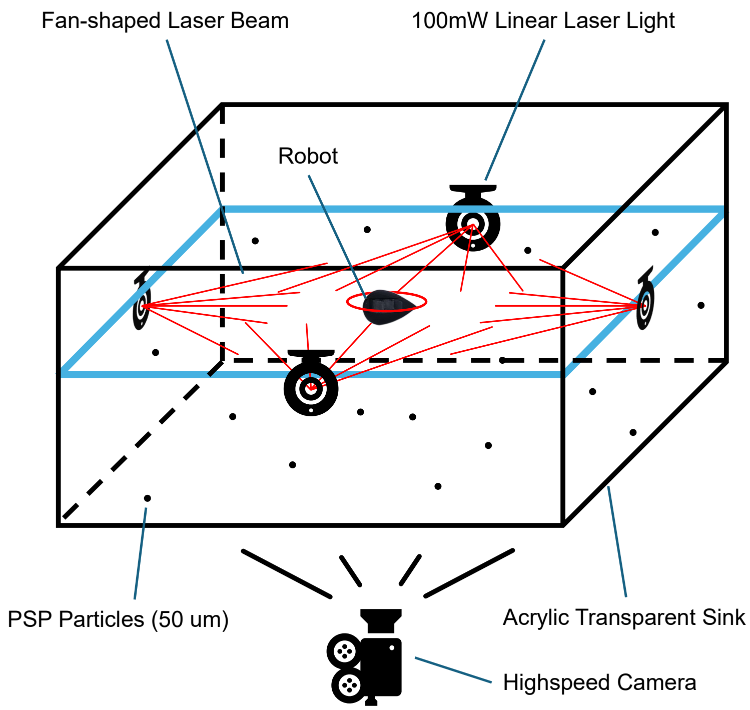

Fig. S17. Testing setup 2: Schematic diagram of the PIV experimental site. Four laser lights illuminate the same underwater horizontal plane where the midpoint of the height of the fins is located. Only the illuminated particles can be recorded by the high-speed camera.

Table S1. Coil parameters

Parameter	Value	Unit
Inner diameter	10.5	mm
Outer diameter	12.5	mm
Length of Coil 1 and Coil 3	6	mm
Length of Coil 2	7	mm
Slot fill factor	0.65	
Electrical resistivity	0.0185	ohm·mm^2/m
Wire diameter	0.15	mm
Number of windings (Coil 1 and Coil 3)	220	
Number of windings (Coil 2)	256	
Permeability of vacuum	1.26×10^{-6}	H/m
Inductance (Coil 1 and Coil 3)	8.8×10^{-4}	H
Inductance (Coil 2)	1.0×10^{-3}	H

Table S2. Magnet parameters

Parameter	Value	Unit
Quantity	2	
Diameter	10	mm
Length	6	mm
Density	0.00196	g/mm^3
Mass	3.7	g
Material	NdFe52	

Table S3. Circuit parameters

Parameter	Value	Unit
Power voltage	5.5	V
Series resistance of the power electronics	1.5	ohm
Resistance of Coil 1 and Coil 3	8.35	ohm
Resistance of Coil 2	9.74	ohm
VCM static resistance	6.15	ohm
VCM current (static stalling)	0.72	A
PWM frequency	10000	Hz

Table S4. Weight budget

Parameter	Value	Unit
VCM	13	g
ERM	1.5	g
PCB	1.2	g
Battery	1.5	g
Shell	6.6	g
Overall	23.8	g
Ratio of magnet weight	0.31	

Movie S1. (.mp4 format). Overview.
Movie S2. (.mp4 format). Tests on granular media.
Movie S3. (.mp4 format). Tests on hard grounds.
Movie S4. (.mp4 format). Jumping height tests.
Movie S5. (.mp4 format). Aquatic tests.
Movie S6. (.mp4 format). System demonstration.
Movie S7. (.mp4 format). Robustness and payload tests.
Movie S8. (.mp4 format). Voice coil motor movies.
Movie S9. (.mp4 format). Simulation movies.
Movie S10. (.mp4 format). PIV movies.
Data S1. (.xlsx format). Collection of experiment data.
